# Associations of activity, sedentary and sleep behaviors with prevalent steatotic liver disease in middle-aged and older adults: the ELSA-Brasil study

**DOI:** 10.1186/s44167-024-00055-7

**Published:** 2024-07-03

**Authors:** Danilo de Paula, Natan Feter, Rodrigo Citton Padilha dos Reis, Rosane Harter Griep, Bruce Bartholow Duncan, Maria Inês Schmidt

**Affiliations:** 1 Department of Epidemiology, Universidade Federal do Rio Grande do Sul, 2400 Ramiro Barcelos St.–2nd floor, Porto Alegre, Rio Grande Do Sul 90035-003 Brazil; 2 Department of Statistics, Universidade Federal do Rio Grande do Sul, 9500 Bento Gonçalves Av Building 43, Porto Alegre, Rio Grande Do Sul 91509-900 Brazil; 3 Laboratório de Educação em Ambiente e Saúde, Instituto Oswaldo Cruz, Fundação Oswaldo Cruz, 1480 Leopoldo Bulhões St., Rio de Janeiro, 21041210 Brazil; 4 Center for Clinical Research, Hospital de Clínicas de Porto Alegre, 2350 Ramiro Barcelos St.-Building 21, 4 Floor, Porto Alegre, Rio Grande do sul 90035-003 Brazil

**Keywords:** Physical activity, Sedentary behavior, Sleep, Steatotic liver (MeSH C06.552.241), Adults, Non-alcoholic fatty liver disease

## Abstract

**Background:**

Steatotic liver disease (SLD) is a prevalent metabolic disease. While single component movement behaviors have been related to its development, comprehensive assessments of their joint associations are scarce.

**Objective:**

To investigate the single-component and multi-component associations of moderate and vigorous physical activity (MVPA), light physical activity (LPA), sedentary behavior (SB), and sleep with prevalent SLD in Brazilian adults.

**Methods:**

A cross-sectional analysis using data from the third wave of the ELSA-Brasil cohort (2017–2019). Participants wore an ActiGraph wGT3X-BT in the waist for seven days and completed a sleep diary. SLD was defined by a Fatty Liver Index ≥ 60. To investigate single-component and multi-component associations, we used three exposure modeling approaches based on Poisson models: multivariable-adjusted regression, restricted cubic splines, and compositional data analysis.

**Results:**

Among 8569 participants (55.7% women, mean age 59.2 ± 8.60), 43.9% had SLD. Total activity volume adjusted for covariates was inversely associated with prevalent SLD. Every 1 mg/day increase in total activity volume was associated with a PR of 0.95 in individuals sleeping < 7 h/day (95% CI 0.94–0.97) and 0.95 (95% CI 0.93–0.96) in those sleeping ≥ 7 h/day. Increasing 30 min/day of MVPA was associated with decreasing prevalence of SLD (sleep ≥ 7 h/day [PR 0.83; 95% CI 0.77–0.89]; sleep ≥ 7 h/day [PR 0.78; 95% CI 0.74–0.83]). Sleep, SB, and LPA were not associated with SLD. Associations of total activity volume and MVPA were more pronounced among females. Adjustment with adiposity markers attenuated the associations.

**Conclusions:**

In adults, total activity volume and MVPA were inversely associated with SLD in a dose–response fashion. Substituting lower-intensity behaviors with MVPA was associated with a lower prevalence of SLD independent of sleep duration, sex, and age.

**Supplementary Information:**

The online version contains supplementary material available at 10.1186/s44167-024-00055-7.

## Background

Steatotic Liver Disease (SLD) [1, 2] is a prevalent liver condition affecting up to 30% of the population [3, 4]. Its underlying pathophysiology concerns increased hepatic and peripheric insulin resistance, which shares common ground with obesity and type 2 diabetes mellitus (T2DM) [5, 6]. Due to its associated complications, such as liver fibrosis, cirrhosis, and liver cancer, it poses a significant global public health problem [7, 8].

The cornerstone of SLD prevention and management relies on lifestyle measures and weight management [7, 9]. For instance, engaging in moderate and vigorous physical activity (MVPA) lowers the risk of mortality in those with SLD by up to 50% [10]. Conversely, prolonged sedentary behavior (SB) is associated with a higher prevalence of the disease [11–14]. Sleep patterns are also associated with SLD, albeit with an unclear nature [15–18].

Studies investigating the associations of device-measured movement behaviors with the occurrence of SLD are scarce [11, 14, 19]. The use of accelerometers allowed for investigations on the effect of movement behaviors on health to consider their multicomponent 24-h pattern rather than single-component behaviors [20–22]. Previous studies on this matter focused mostly on single-component movement behaviors, often with limited sample sizes and restricted to Asian and European populations [11, 14, 19]. Furthermore, the influence of sleep duration, age, and sex in these associations has been little explored. In this sense, the large sample size of the ELSA-Brasil study allows for the investigation of possible modifiers of these associations. It also enables the evaluation of the dose–response gradient for movement behaviors and SLD, as well as the theoretical effects of exchanging behaviors, which can help the design of effective interventions.

Thus, our cross-sectional study aims at producing a comprehensive assessment of the association of 24-h movement behaviors with SLD by investigating (1) the dose–response associations of single-component device-measured movement behaviors with SLD; (2) the association of the composition of movement behaviors with SLD prevalence; (3) which theoretical substitutions between movement behaviors could yield maximal benefit in reducing the prevalence of SLD; and (4) the influence of age, sex, and sleep duration on these associations.

## Methods

### Study design

The Estudo Longitudinal de Saúde do Adulto (Longitudinal Study of Adults’ Health - ELSA-Brasil) is a multicenter cohort investigating noncommunicable disease risk factors in Brazilian adults [23, 24]. After a wide and open invitation to potential participants, we enrolled 15,105 active and retired civil servants of all ranges of occupations from public research, education, and healthcare organizations in six Brazilian states.

At baseline (2008 to 2010), volunteers were aged 35–74. Participants signed an informed consent form, underwent a thorough health assessment, and provided blood and urine samples [25, 26]. For this paper, we analyzed cross-sectional data from participants of wave 3 (2017 to 2019), encompassing 12,636 eligible participants (550 deceased and 1919 lost during follow-up).

### Measurements

We obtained overnight 12-h fasting blood samples through venipuncture and conducted a 2-h 75 g oral glucose tolerance test (OGTT) in participants without a prior diagnosis of diabetes [27]. Plasma glucose, glycated hemoglobin (HbA1c), aspartate aminotransferase, alanine transaminase, gamma-glutamyl transferase, triglycerides, and high density lipoprotein were measured in a centralized laboratory [28].

Anthropometric data were collected using a fixed stadiometer (Seca 216, Seca Brasil, Cotia-SP, Brazil) for height and a bioelectrical impedance analyzer (BIA; Inbody230, InBody Co., Ltd., Seoul, Korea) with 8-point tactile electrodes for weight and body composition. Body Mass Index (BMI) was calculated as weight divided by height squared (kg/M1), and body fat percentage was determined as the total fat mass divided by participants’ weight [29]. Waist circumference was the average of two measures between the right iliac crest and the 12th right rib using an inelastic measurement tape.

Blood pressure was measured three times using an oscillometer device (Omron HEM 705CPINT) on the right arm in a quiet room with controlled temperature (20–24 °C) after 5 min of sitting rest [25]. Diabetes classification included previous physician diagnosis; glucose-lowering medication use in the last two weeks (cross-verified against prescriptions) or one laboratory abnormality: fasting plasma glucose ≥ 126 mg/dL (7.0 mmol/L) or 2 h post-load plasma glucose ≥ 200 mg/dL (11.1 mmol/L) or glycated hemoglobin ≥ 6.5% (48 mmol/mol). [30, 31] Certified technicians conducted all measurements following standardized protocols [24].

### Movement behaviors

Time in MVPA, LPA, and SB were assessed over 7 days using a waist-worn ActiGraph GT3X + (version 3.2.1, Pensacola, USA). Participants were instructed to wear the devices throughout the 24 h of the day, only removing them when doing activities involving water. We averaged participants’ daily movement behaviors across all valid days of participants with validated wear (≥ 4 days, ≥ 16 h/day) [32–34]. We determined the triaxial summary acceleration as the average Euclidean Norm Minus One (ENMO = $\sqrt{{x}^{2}+{y}^{2}+ {z}^{2}}- 1g$) for each 5-s epoch. Total activity volume was calculated as the average acceleration during wear. Movement behaviors were defined according to validated thresholds. Specifically, MVPA was characterized by acceleration > 69 mg, LPA 69–15 mg, and SB < 15 mg during wake time [35]. Sleep was calculated using a sleep diary filled by the participant during the same week of the accelerometer use. We then calculated the difference in minutes from the sleep time to the next waking time [36–38]. Details an these procedures were previously published [38]. Movement behavior durations were described as arithmetic means, standard deviations (SD), and geometric means.

### SLD

We defined SLD as a Fatty Liver Index (FLI) ≥ 60, a composite index including BMI, waist circumference (WC), triglycerides (TG), and gamma-glutamyl transferase (γGT) to predict steatotic liver. The index was calculated as shown below [39].

$${\varvec{F}}{\varvec{L}}{\varvec{I}}=\boldsymbol{ }\frac{{({\varvec{e}}}^{0.953\boldsymbol{*}{\varvec{l}}{\varvec{o}}{\varvec{g}}{\varvec{e}}\left({\varvec{T}}{\varvec{G}}\right)\boldsymbol{ }+\boldsymbol{ }0.139\boldsymbol{*}{\varvec{B}}{\varvec{M}}{\varvec{I}}+0.718\boldsymbol{*}{\varvec{l}}{\varvec{o}}{\varvec{g}}{\varvec{e}}\left({\varvec{\gamma}}{\varvec{G}}{\varvec{T}}\right)\boldsymbol{ }+\boldsymbol{ }0.053\boldsymbol{*}{\varvec{l}}{\varvec{o}}{\varvec{g}}{\varvec{e}}({\varvec{W}}{\varvec{C}})-15.745\boldsymbol{ }})}{{(1+\boldsymbol{ }{\varvec{e}}}^{0.953\boldsymbol{*}{\varvec{l}}{\varvec{o}}{\varvec{g}}{\varvec{e}}\boldsymbol{ }\left({\varvec{T}}{\varvec{G}}\right)\boldsymbol{ }+\boldsymbol{ }0.139\boldsymbol{*}{\varvec{B}}{\varvec{M}}{\varvec{I}}+0.718\boldsymbol{*}{\varvec{l}}{\varvec{o}}{\varvec{g}}{\varvec{e}}\boldsymbol{ }\left({\varvec{\gamma}}{\varvec{G}}{\varvec{T}}\right)\boldsymbol{ }+\boldsymbol{ }0.053\boldsymbol{*}{\varvec{l}}{\varvec{o}}{\varvec{g}}{\varvec{e}}({\varvec{W}}{\varvec{C}})-15.745\boldsymbol{ }})}\boldsymbol{*}100$$

Validated against abdominal ultrasound, it achieved an area under the curve (AUC) receiver operator characteristic (ROC) of 0.85 (95% CI 0.82 to 0.88). A score of ≥ 60 yielded a positive likelihood ratio (+ LR) of 4.3 [39]. The instrument was assessed against ultrasound in the ELSA-Brasil sample, reaching an area under the curve receiver operator characteristic of 0.82 (95% CI 0.8 to 0.84) and a positive likelihood ratio of 2.93 for moderate steatosis using the threshold of 60 [40].

### Covariates

Covariates included study center, age, sex, degree of schooling, household income, employment status (working/retired), smoking status (never, former, current), use of antihypertensive, cholesterol-lowering, and glucose-lowering medication. Total energy intake, estimated from a validated food frequency questionnaire [41], and alcohol consumption in milliliters per week were also considered. Alcohol use was categorized as never, former, or current, with a description of frequency and volume of consumption. Excessive alcohol consumption was defined as ≥ 140 g/week for females and ≥ 210 g/week for males [7, 9].

Race/color was self-declared and grouped using pre-defined categories reflecting sociodemographic similarities particular to the Brazilian population. Individuals identifying as “yellow” (Asian descent), due to small numbers (n = 208), were grouped with white individuals given their socioeconomic similarities [42]. Similarly, “indigenous” (n = 65) were analyzed alongside black and brown individuals, considering their shared historic social vulnerability in Brazil [43].

### Statistical analyses

We utilized robust Poisson regression to investigate the associations of single-component movement behaviors with prevalent SLD, estimating prevalence ratios (PR). Cubic splines of single-component movement behaviors were constructed to assess whether the association expressed a linear dose–response relationship or reached a plateau in prevalence reduction. We placed knots at each group’s 10th, 50th, and 90th percentiles, setting the lowest observed exposure level as the reference category for total activity volume and MVPA, and the 5% percentile for LPA and SB. For sleep, the reference was set at 7 h based on international recommendations [44]. We employed compositional data analysis (CoDA) to evaluate the association of the 24-h composition of movement behaviors with the prevalence of SLD [45–48]. Using compositional models, we estimated the change in SLD prevalence with reallocations of time between behaviors through isotemporal substitution predictions [45, 49]. Supplementary methods are provided in the additional file, along with detailed information on the procedures. Models were adjusted as follows: Model 1 (fully adjusted) study center, age, sex, race/color, degree of schooling, income quintiles, employment, smoking status, alcohol consumption, hypertension, diabetes, and total energy intake. Because BMI might mediate the association between movement behaviors and SLD, our primary analyses are presented without adjustment for BMI. However, adiposity may confound this association, so alternative models are presented including BMI (Model 2a) and percent body fat (%BF) (Model 2b) to Model 1. We chose percent body fat as an alternative measure to mitigate the risk of overfitting the models due to the presence of BMI in defining the outcome (FLI ≥ 60).

We presented results stratified based on achieving more or less than 7 h/day of sleep [22, 50]. In addition, we assessed age and sex as possible modifiers given previous knowledge. Briefly, physical activity and sedentary behavior play a central role in insulin resistance, which increases with aging and leads to metabolic diseases [51]. Furthermore, as individuals age, they tend to develop a less active and more sedentary movement behavior profile [38], which also raises the risk of metabolic diseases. We also evaluated the role of sex in the associations since women usually have less active movement behaviors [38], while men more often develop metabolic diseases. Finally, physiological variability related to age and sex, such as body fat distribution and hormonal differences [52], may also alter the movement behaviors associations with SLD. To assess these potential effect modifications we tested interactions for movement behaviors with sex and age by additionally inserting terms of each ILR with the potentially modifier variables in the models. We considered the interaction worth further investigation if their p-value was lower than 0.2.

Sensitivity analyses included (1) subclassifying SLD in metabolic dysfunction-associated steatotic liver disease (MASLD) using the consensus diagnostic criteria [2] (Supplementary methods, additional file); (2) excluding 534 participants with excessive alcohol consumption that would be classified as metabolic-alcoholic steatotic liver disease (MetALD). Figures for the stratified analysis and sensitivity analyses are provided in the Supplementary file).

We set the significance level at p < 0.05 (two-sided), and estimates are reported with 95% confidence intervals (95% CI). Convergence of the Poisson models was evaluated, and residuals were visually inspected. Collinearity was assessed using the Generalized Variance Inflation Factor (GVIF); a threshold of 2.5 was considered possible collinearity. All data processing and analyses were conducted using the R software version 4.2.1. (R Core Team, 2021, The R Foundation for Statistical Computing, Vienna, Austria). We reported the results according to the STROBE Statement [53]. The checklist is available in the additional file.

## Results

**Fig. 1:**
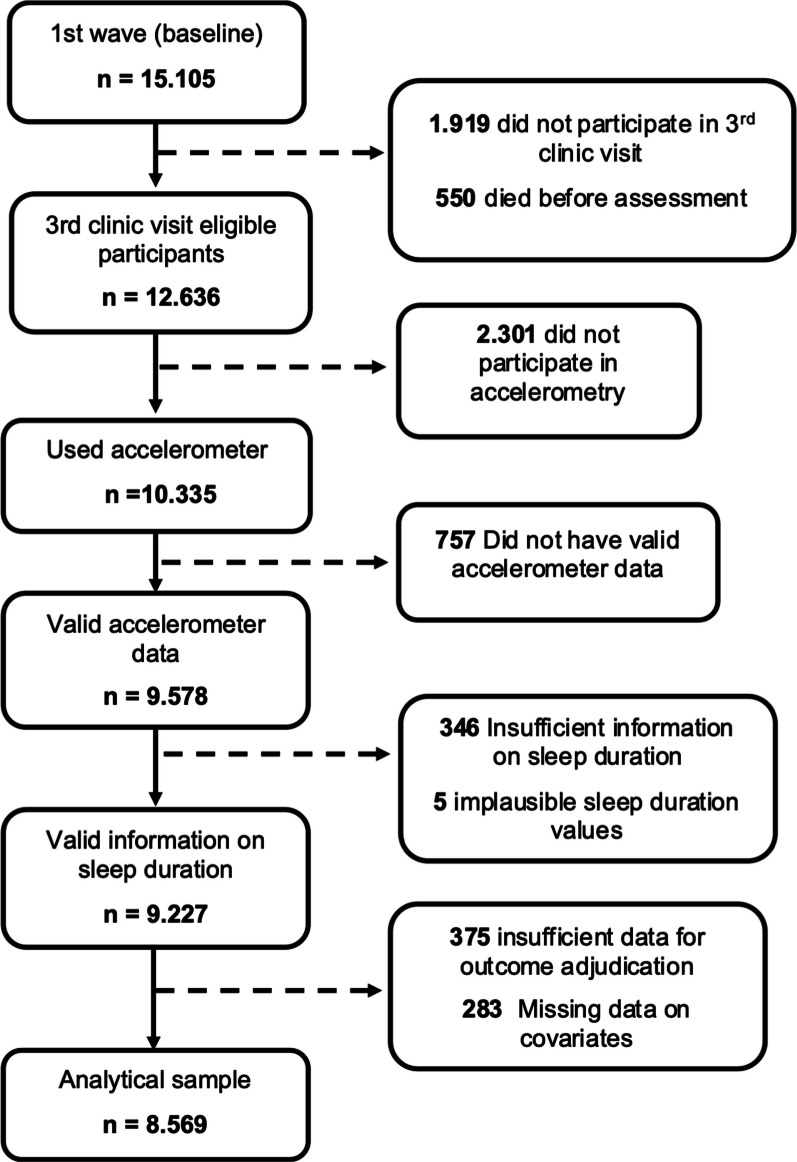
Flow chart of the sample ELSA-Brasil study wave 3 (2017–2019)

After excluding participants with missing data, the analytical sample included 8569 subjects (Fig. 1) with a mean age of 59.2 (8.6) years. As shown in Table 1, there were 3764 (43.9%) prevalent cases of SLD, with higher prevalence among men (p < 0.001), black subjects (p < 0.001), and those with lower education (p < 0.001) and income (p < 0.001).

**Table 1:** Participant’s characteristics according to presence of steatotic liver disease, ELSA-Brasil study (2017–2019), n = 8569

	SLD^a^ n = 3764	No SLD^a^ n = 4805	Overall^b^ n = 8569	
Age-years (SD)	59.2 (8.18)	59.2 (8.93)	59.2 (8.61)	
Sex				
Female	1731 (36.3%)	3044 (63.7%)	4775 (55.7%)	
Male	2033 (53.6%)	1761(46.4%)	3794 (44.3%)	
Degree of schooling				
Less than high school	435 (57.2%)	326 (42.8%)	761 (8.88%)	
High school	1298 (49.7%)	1312 (50.3%)	2610 (30.5%)	
University degree	2031 (39.1%)	3167 (60.9%)	5198 (60.7%)	
Race/color^c^				
Black	615 (50.2%)	610 (49.8%)	1225 (14.3%)	
Brown	1112 (46.4%)	1286 (53.6%)	2398 (28.0%)	
White	1944 (41.6%)	2729 (58.4%)	4673 (54.5%)	
Asian descent	56 (1.49%)	152 (3.16%)	208 (2.4%)	
Indigenous Brazilian	37 (0.98%)	28 (0.58%)	65 (0.8%)	
Income quintile (BRL range)				
1st (71–1410)	862 (53.0%)	763 (47.0%)	1625 (19.0%)	
2nd (1410–2340)	863 (46.7%)	986 (53.3%)	1849 (21.6%)	
3rd (2340–3560)	704 (42.7%)	944 (57.3%)	1648 (19.2%)	
4th (3560–5930)	760 (40.4%)	1119 (59.6%)	1879 (21.9%)	
5th (5930–17800)	575 (36.7%)	993 (63.3%)	1568 (18.3%)	
Working status				
Currently working	2841 (44.8%)	3496 (55.2%)	6337 (74.0%)	
Retired	923 (41.4%)	1309 (58.6%)	2232 (26.0%)	
Smoking status				
Never	2122 (40.4%)	3131 (59.6%)	5253 (61.3%)	
Former	1321 (51.4%)	1249 (48.6%)	2570 (30.0%)	
Current	321 (43.0%)	425 (57.0%)	746 (8.71%)	
Use of alcohol				
Never	395 (40.2%)	589 (59.8%)	982 (11.5%)	
Former	884 (44.5%)	1104 (55.5%)	1988 (23.2%)	
Current	2483 (44.4%)	3111 (55.6%)	5594 (65.3%)	
Excessive alcohol consumption—n	534 (56.7%)	408 (43.3%)	942 (11.0%)	
Alcohol consumption—gr/week (SD)	86.0 (159)	55.1 (106)	68.7 (133)	
Daily energy intake—kcal/day (SD)	2552 (1086)	2296 (934)	2408 (1011)	

Continuous variables are reported as means (standard deviations), and categorical variables as frequencies (percentage)

SLD, steatotic liver disease; a = percentages correspond to the lines, b = percentages correspond to the columns; c = self-reported and classified according to the Brazilian Agency for Geography and Statistics (IBGE)

Individuals with SLD had more metabolic dysfunctions, higher BMI, and a higher prevalence of T2DM (Supplementary Table 1). On average (SD) time engaged in MVPA, LPA, SB, and sleep were respectively 47.4 (25.3) minutes/day, 207 (67.8) minutes /day, 736.8 (99.6) minutes /day, and 448.2 (71.4) minutes /day (Supplementary Table 2). Crude movement distribution differences between participants with and without SLD were minimal (Supplementary Fig. 1).

### Single-component behaviors and SLD

**Fig. 2:**
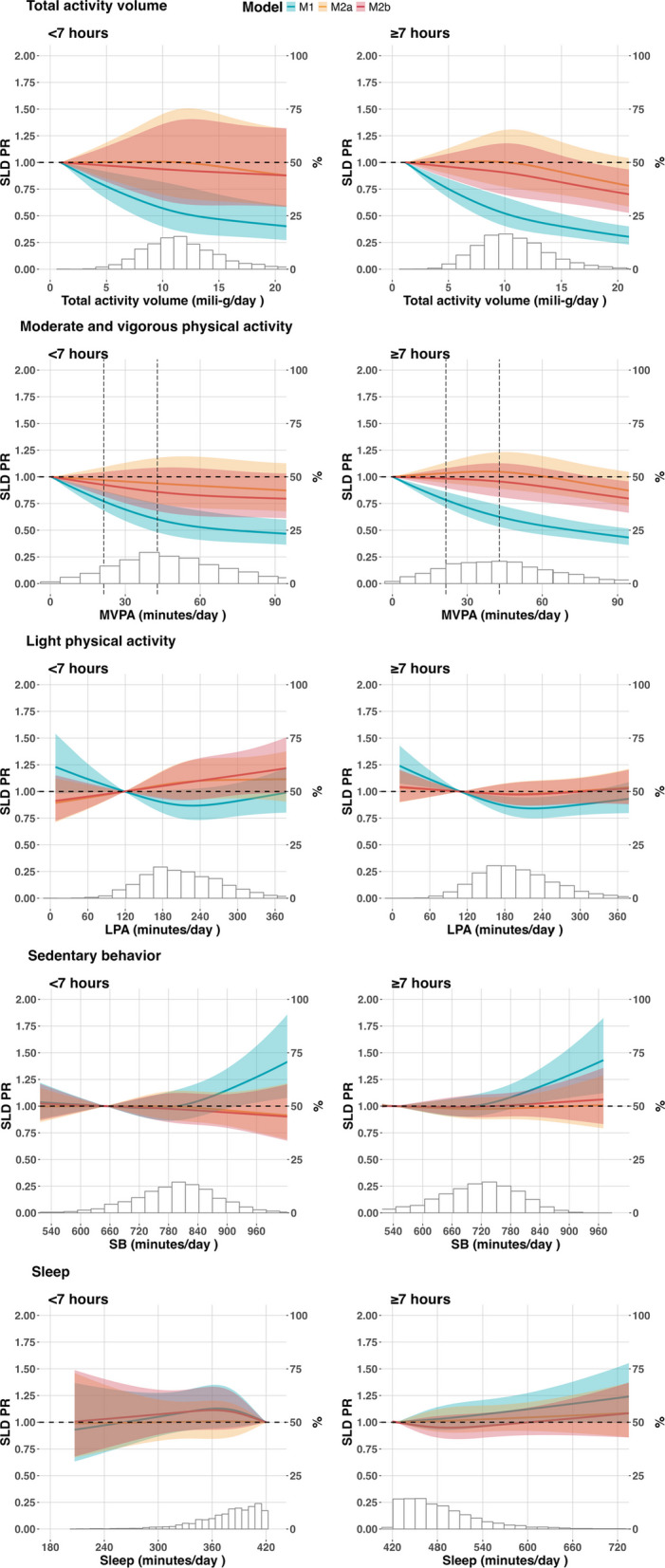
Dose–response associations of physical activity with steatotic liver disease by sleep duration, ELSA-Brasil (2017–2019), n = 8569. Prevalence Ratios (PR) for Steatotic liver disease (SLD) were estimated with Poisson regression models using restricted cubic splines with knots placed at 10th, 50th, and 90th percentiles of the exposure and adjusted for—M1 (blue): study center, sex, age, race/color, income, degree of schooling, smoking, alcohol consumption, diabetes, hypertension, and daily energy intake; M2a (yellow): M1 and further adjustment for body mass index. M2b (red): M1 and further adjustment for % body fat. Continuous lines are the point estimates of PR across the exposure spectra, and the colored hatched area is the 95% confidence interval. The histograms show the sample distribution on the exposure spectra, with the right vertical axis showing the percentage of the study sample. MVPA, moderate-and-vigorous physical activity

Table 2 shows the associations of total activity volume and movement behaviors with prevalent SLD. A 1mili-*g* increase in total activity volume was linked to a 3 to 4% lower prevalence of SLD (model 1—sleep < 7 h/day: PR 0.97, 95% CI 0.95 to 0.98; sleep ≥ 7 h/day: PR 0.96, 95% CI 0.95 to 0.98). Decreasing PRs of SLD were observed across the entire range of total activity volume, with a steeper decline from 0 to 10 mg/day (Fig. 2A and B). Incorporating adiposity measures nullified associations in short sleepers. Non-short sleepers had a lower prevalence of SLD with total activity volume exceeding 17 milli-*g*/day after adjusting for %BF (Model 2b) but not for BMI (Model 2a).

**Table 2:** Association of device-measured movement behaviors and prevalent steatotic liver disease. ELSA-Brasil study (2017–2019), n = 8569

Model	Total activity volume 1 mg/day *PR (95% CI)*	MVPA 30 min/day *PR (95% CI)*	LPA 30 min/day *PR (95% CI)*	SB 30 min/day *PR (95% CI)*	Sleep 30 min/day *PR (95% CI)*
Sleep < 7 h					
M1	0.97 (0.95; 0.98)	0.85 (0.80; 0.92)	1.00 (0.98; 1.03)	1.01 (0.99; 1.03)	1.01 (0.97; 1.06)
M2a	0.99 (0.97; 1.01)	0.96 (0.89; 1.03)	1.01 (0.99; 1.04)	1.00 (0.98; 1.01)	1.00 (0.95; 1.05)
M2b	0.99 (0.98; 1.01)	0.95 (0.88; 1.02)	1.02 (1.00; 1.05)	0.99 (0.97; 1.01)	0.99 (0.94; 1.04)
Sleep ≥ 7 h					
M1	0.96 (0.95; 0.97)	0.83 (0.78; 0.87)	0.99 (0.98; 1.01)	1.01 (1.00; 1.02)	1.01 (0.99; 1.03)
M2a	0.99 (0.98; 1.00)	0.95 (0.90; 1.01)	1.00 (0.99; 1.02)	1.00 (0.99; 1.01)	1.01 (0.99; 1.03)
M2b	0.99 (0.97; 1.00)	0.93 (0.88; 0.98)	1.00 (0.99; 1.02)	1.00 (0.99; 1.02)	1.00 (0.98; 1.02)

SLD, Steatotic liver disease; PR, Prevalence rate ratios, CI, confidence interval, MVPA, moderate to vigorous physical activity, LPA, light physical activity, SB, Sedentary behavior, Model 1, adjusted for study center, sex, age, race/color, income, degree of schooling, smoking, alcohol consumption, diabetes, hypertension, and daily energy intake; Model 2a, Model 1 plus body mass index; Model 2b, Model 1 plus % body fat

Inverse associations between MVPA and SLD were observed regardless of sleep duration (Table 2). Additional 30 min/day of MVPA were associated with PRs of 0.85 (95% CI 0.79 to 0.92) in short sleepers and 0.83 (95% CI 0.78 to 0.87) in non-short sleepers. As depicted in Fig. 2, PRs declined throughout the entire MVPA spectrum. Associations were eliminated among short sleepers when adiposity measures were added. (Fig. 2). In non-short sleepers, adjusting for BMI (Model 3a) nullified associations, while adjusting for %BF (Model 3b) attenuated the association. In this group, the %BF model showed a lower SLD prevalence only with high engagement on MVPA, approximately 70 min/day (Fig. 2D).

We did not observe a significant association of LPA, SB, and sleep duration with the prevalence of SLD in the adjusted models. Table 2 However, in the splines, the group sleeping ≥ 7 h a day showed a slightly lower prevalence of SLD with higher LPA. Additionally, both sleep duration groups expressed a higher prevalence of SLD with very high engagement in sedentary behavior. All these results vanished with adjustment for body fat markers Fig. 2.

### Compositional isotemporal substitution and SLD

**Fig. 3:**
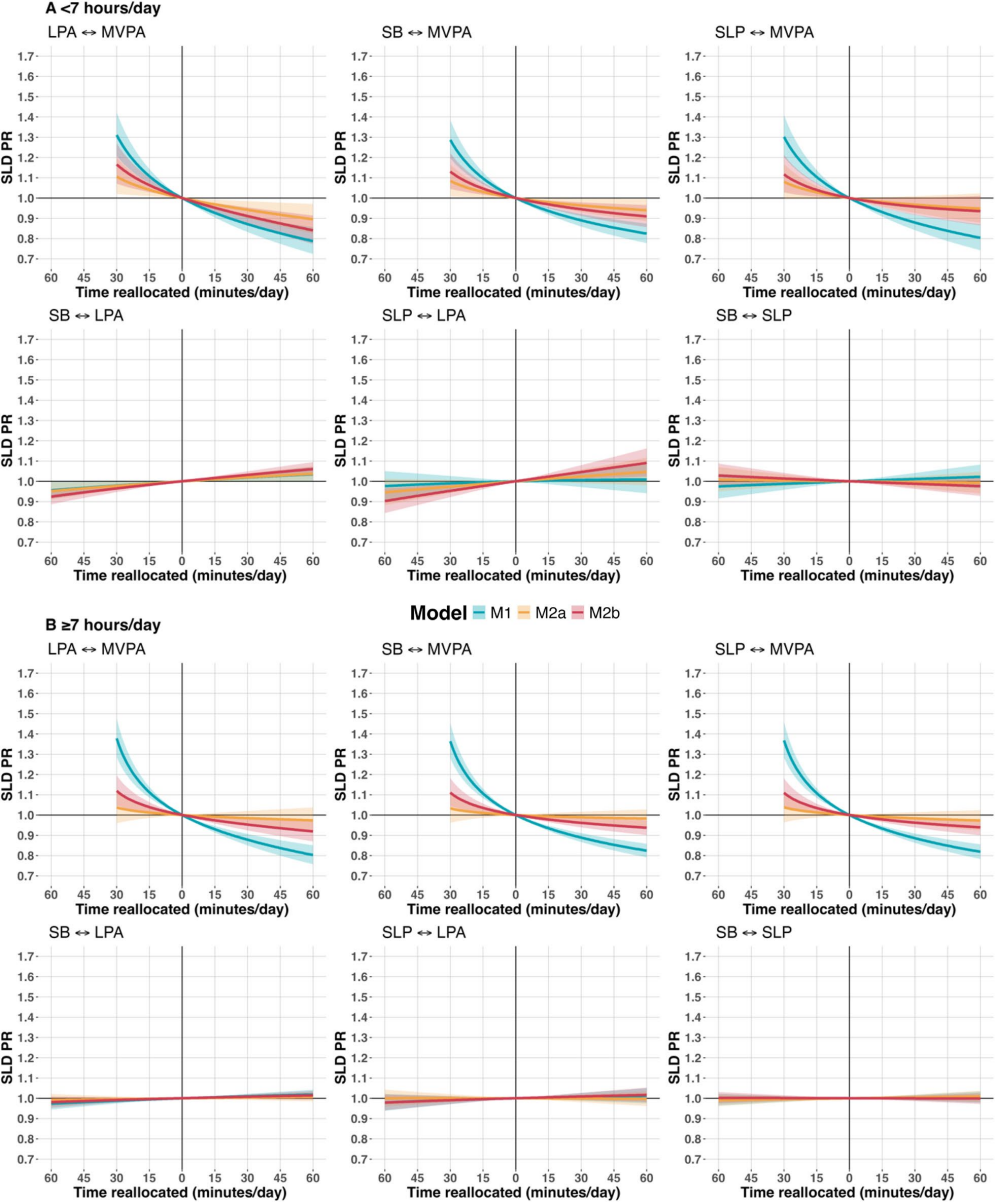
Exchanging movement behaviors association with prevalent steatotic liver disease by sleep duration ELSA-Brasil (2017–2019), n = 8569. Plots show the predicted prevalence ratios (PR) of Steatotic liver disease (SLD) resulting from reallocating time between movement behaviors using compositional isotemporal substitution robust Poisson models. The vertical continuous black line (x = 0) represents the mean of behaviors of the group as the reference—A = Sleep < 7 h: MVPA: 44.92 min/day, LPA: 209.0 min/day, SB: 803.8 min/day, SLP: 382.2 min/day; B = Sleep ≥ 7 h: MVPA: 38.2 min/day, LPA: 194.8 min/day, SB: 714.0 min/day, SLP: 493.0 min/day. The two behaviors not shown in each plot have their values fixed at the geometric mean for the analysis group. MVPA = moderate and vigorous physical activity; LPA = light physical activity; SB = sedentary behavior. Models were adjusted as follows—Model 1 (M1, blue): study center, sex, age, race/color, income, degree of schooling, smoking, alcohol consumption, diabetes, hypertension, and daily energy intake; Model 2a (M2a, yellow): M1 and further adjustment for body mass index. Model 2b (M2b, red): M1 and further adjustment for % body fat

Figure 3 illustrates the theoretical impacts of bidirectionally reallocating time between movement behaviors. Among short sleepers, substituting LPA or SB with MVPA was linked to a lower prevalence of SLD, regardless of BMI or %BF adjustments. Moreover, reallocating sleep to MVPA was associated with a lower prevalence of SLD with the adjusted model (Model 1) but not after incorporating body adiposity (Models 2a and 2b). For non-short sleepers, reallocating as little as 1 min from other MBs to MVPA was associated with decreased prevalence of SLD irrespective of including %BF, but not with adjustment for BMI. Conversely, reducing MVPA in favor of other movement behaviors was associated with a raised prevalence of SLD in all models. For instance, exchanging 30 min of SB for MVPA resulted in an 11% lower prevalence of SLD (Model 1 – sleep < 7 h/day 0.89 [95% CI 0.86 to 0.92] and sleep ≥ 7 h/day PR 0.89 [95% CI 0.87 to 0.91]), while swapping 30 min of MVPA with SB was associated with a 20 to 37% higher prevalence of SLD (sleep < 7 h/day PR 1.29 [95% CI 1.20 to 1.39]; sleep ≥ 7 h/day PR 1.37 [95% CI 1.28 to 1.46]). No significant associations were observed in the bidirectional substitutions between other movement behaviors.

### Subgroup analyses

There was little evidence for a relevant interaction between age and the composition of movement behaviors in relation to the prevalence of SLD (p > 0.2). We observed an interaction between movement behavior composition and sex (p < 0.01), revealing stronger associations in females compared to males (Supplementary Tables 3 and 4). Associations were attenuated in all groups upon adjusting for adiposity measures.

In females sleeping ≥ 7 h/day, when including %BF, a lower prevalence of SLD was observed starting from 16.5 mg/day of total activity volume, the higher 5% of the sample distribution (Supplementary Fig. 2). The same trend was noted for MVPA, with females showing a more substantial association than males (Supplementary Tables 3 and 4). Sex-specific cubic splines are presented in Supplementary Figs. 2 and 3. In compositional models (Supplementary Figs. 4 and 5), females exhibited a more pronounced decrease in the prevalence of SLD with increasing MVPA at the cost of any other MB compared to males. Furthermore, individuals from both sexes in the group with sleep ≥ 7 h/day showed a larger decrease in the prevalence of SLD with increasing MVPA. Lastly, including BMI in the models eliminated associations, while adjustment for %BF did not eliminate associations in males sleeping ≥ 7 h/day. (Supplementary Figs. 4 and 5).

### Sensitivity analyses

When applying the SLD subclassification of MASLD, we identified 3762 participants with the condition. Both sensitivity analyses were consistent with the previous results. (Supplementary tables 5 & 6 and Supplementary Figs. 6 and 7). The analysis excluding the 534 participants with high alcohol consumption (187 in the short sleep and 347 in the non-short sleep group) produced results aligning with the primary analysis, demonstrating a larger association magnitude. Notably, in this scenario, including adiposity markers did not eliminate the associations. (Supplementary Table 7 and Supplementary Figs. 8 and 9).

## Discussion

Our cross-sectional analyses of a large, multicenter, and ethnically diverse sample of adults explored the dose–response and compositional associations of device-measured movement behaviors with Steatotic Liver Disease. We observed that a 1 mg/day higher daily total activity volume was linked to a 5% lower prevalence of SLD. Previous studies suggest that 1 mg/day measured with wrist accelerometers translates to approximately 500 steps or 5 min of brisk walking a day [54–57]. Considering the lower acceleration magnitude of hip-worn accelerometers compared to wrist-worn accelerometers [58], it is reasonable to affirm that this value is somewhat higher for measurements in the hip, although no study has directly assessed this question.

Regarding movement behaviors, MVPA—but not LPA, SB, or sleep—exhibited a dose–response association with the prevalence of SLD regardless of sex and sleep duration. However, adjusting for %BF or BMI nullified associations for single-component behaviors. On the other hand, a theoretical increase in time in MVPA at the cost of any other behavior—LPA, SB, or sleep—was associated with a progressively lower prevalence of SLD. Interestingly, reducing MVPA by increasing the other MBs was linked to an asymmetrically higher prevalence of SLD.

Our study aligns with previously described inverse associations of total activity volume and MVPA with the prevalence of SLD, validating results obtained with self-reported and device-based PA measures [13, 14, 59–64]. Despite a well-established association, little evidence of a dose–response relationship was available before this study. Nonetheless, a similar study with a Japanese sample found a steep decline in the odds of ultrasound-measured SLD up to 300 weekly minutes (1800 MET-min/week) of device-measured MVPA [14]. Our diverse sample provides new evidence of a dose–response association between MVPA and SLD while considering the non-linear relationship between sleep and SLD and assessing the influence of sex, age, and sleep groups. Our findings also demonstrate a significant decrease in SLD prevalence throughout the whole spectra of exposure to MVPA. We observed a steeper decline in the lower range of exposure up to approximately 420 weekly minutes (60 min/day) of MVPA, supporting the World Health Organization's updated recommendation of 150 to 300 weekly minutes of moderate activity [65]. We still observed a decline in prevalence with exposure higher than 60 min a day, however, its magnitude was smaller than in lower exposure levels. Incorporating markers of adiposity (BMI or %BF) into our model attenuated all associations. However, total activity volume and MVPA associations with SLD and the substitutions remained significant when adjusting for %BF and vanished with BMI adjustment. Given that BMI is a component of the Fatty Liver Index, we argue that %BF adjustments provide better control for adiposity-related confounding. Whether this factor plays the role of a confounder or a mediator in such an association remains an unclear question. However, our analyses suggest that the association between MVPA and SLD is independent of adiposity, with exercise interventions supporting a direct mechanism for this association [66].

Despite participants spending more time in LPA than MVPA, our study did not find an association between LPA and SLD in individuals sleeping less than 7 h, but a slightly lower prevalence of SLD with higher LPA in those sleeping ≥ 7. Three recent cross-sectional studies using device-measured LPA showed inconsistent findings, with two describing an inverse association [11, 19] and a third not observing an association [14]. The evidence suggests that the volume and intensity of activity are essential for preventing this condition, but inconsistencies in cross-sectional studies necessitate further prospective investigations. This hypothesis is corroborated by clinical trials showing a beneficial effect of at least moderate intensity training in liver fat content [67] and an unclear effect of lower intensity modalities [68, 69].

Contrary to our expectations, we observed a higher prevalence of SLD only with very high levels of SB, aligning with other investigations that described a higher prevalence of SLD in the most sedentary participants [13, 63, 70]. One previous similar study did not observe this association [14]. Meanwhile, isotemporal substitution models revealed a direct association of SB with SLD if replaced by MVPA. Factors that explain the inconsistency across studies are a high amount of time in SB (12 h/day) and a small amount of MVPA (3% of the day) in our sample. Considering we observed a higher prevalence of SLD when substituting MVPA with SB, the associations other studies described could have resulted from even lower MVPA levels rather than higher SB levels. Also, the previous study investigating dose–response associations of SB with SLD did not consider sleep duration, which may have distorted associations as sleep duration is likely to have a non-linear association with SLD [15–17].

In our compositional analyses, we found that replacing any behavior with MVPA was associated with a decreased prevalence of SLD. Conversely, exchanging MVPA with any other movement behavior was associated with an increased prevalence of SLD, with varying magnitudes. Such results emphasize the importance of considering the total 24-h distribution of movement behaviors in health research, aligning with previous studies. For instance, studies using device-based and self-reported measures showed that replacing SB or LPA with MVPA lowered SLD odds while reallocating time from MVPA to SB or LPA increased SLD odds [14, 64]. Another study in rural Chinese older adults found lower SLD odds by substituting SB with LPA, not SB with MVPA, likely due to high LPA levels in their sample [11]. These findings enhance our understanding of how different movement behaviors influence SLD risk.

The results of the sensitivity analyses were highly consistent with the primary analysis. Exclusion of participants with MASLD and high alcohol consumption, defined as MetALD, reinforced the theoretical framework linking physical activity to metabolic diseases through insulin resistance improvement.

Our study has its limitations. The cross-sectional design limits causal inferences about the association, and its observational component produces the risk of bias due to unmeasured confounders. However, given the absence of longitudinal evidence and the lack of large, well-conducted clinical trials, our study enhances the understanding of the relationship between movement behaviors and the presence of SLD. Secondly, we could not evaluate SB’s postural component, so our operational definition of SB differs from the formal one [71]. However, we used validated thresholds to identify SB, which should address this issue. Also concerning movement behaviors, due to a lack of validated algorithms to identify sleep duration from raw accelerometer data, we have used self-reported measures for this movement behavior. While this approach may be susceptible to measurement bias due to its subjective nature, the detailed sleep diary used was filled during the same time of the accelerometer use, minimizing recall bias Thirdly, we defined SLD using serological markers. Nonetheless, the Fatty Liver Index was validated with satisfactory diagnostic properties and is extensively used in epidemiological research [39, 40]. In regards to the statistical analysis, because our outcome was defined by using the FLI, which includes BMI, adjustment for BMI may generate overfitting of the models. We alternatively adjusted the analyses for %BF, a validated measure of adiposity, to address this issue [29]. Lastly, the multiple testing conducted may raise concerns about type 1 errors.

Our study has notable strengths. Firstly, the ELSA-Brasil study is composed of a highly diverse sample regarding race/ethnicity, cultural, and socioeconomic background. Thus, albeit corroborating previous studies, we provide new insights by enhancing the generalizability to other locations and populations. Secondly, our results are highly reliable due to meticulously standardized data collection and stringent quality control procedures [25, 26]. Additionally, our use of 7-day accelerometry, a more precise approach than questionnaires to assess PA, is uncommon in large epidemiologic studies of low and middle-income countries. Thirdly, our sample size allowed for subgroup analyses, exploring potential effect modifiers such as sleep duration, age, and sex, a novel contribution. Lastly, we used the updated definition of SLD, aligning more closely with the pathophysiological framework and being defined with positive diagnostic criteria rather than using a diagnosis of exclusion.

## Conclusion

In conclusion, total activity volume and MVPA were associated with SLD. MVPA exhibited a dose–response relationship, with a decreasing prevalence of SLD up to 420 min per week, aligning with current recommendations. Our compositional analysis highlights that any increase in MVPA correlates with a lower prevalence of SLD regardless of age, sex, sleep duration, and replaced behavior. The findings underscore the message that “every move counts” and emphasize the importance of considering the full 24-h distribution in health studies. Notably, this framework is pivotal when designing lifestyle interventions to address the growing burden of non-communicable diseases at the individual and population levels.

## Supplementary Information

Supplementary file 1.

## Data Availability

Due to ethical restrictions approved by the ethics committee of each institution (Universidade Federal de Minas Gerais, Universidade de São Paulo, Universidade Federal do Espírito Santo, Universidade Federal do Rio Grande do Sul, Universidade Federal da Bahia e Fundação Oswaldo Cruz) and by the Publications Committee of ELSA-Brasil (publiELSA), the data used in this study can be made available for research proposals by a request to ELSA-Brasil's Data Center (estatisticaelsa@gmail.com) and to ELSA-Brasil's Publications Committee. Additional information can be obtained from the ELSA-Brasil Coordinator of the Research Center of Rio Grande do Sul (maria.schmidt@ufrgs.br).

## Abbreviations

+ LR: Positive likelihood ratio
%BF: Percent body fat
AUC: Area under the curve
BMI: Body mass index
CI: Confidence interval
ELSA-Brasil: Estudo Longitudinal de Saúde do Adulto
FLI: Fatty liver index
FPG: Fasting plasma glucose
GVIF: Generalized Variance Inflation Factor
HbA1c: Glycated haemoglobin
HDL: High density lipoprotein
LPA: Ligth physical activity
MASLD: Metabolic dysfunction associated steatotic liver disease
MB: Movement behaviors
MetALD: Metabolic-alcoholic steatotic liver disease
MVPA: Moderate and vigorous physical activity
NAFLD: Nonalcoholic fatty liver disease
OGTT: Oral glucose tolerance test
PR: Prevalence ratios
ROC: Receiver operator characteristic
SB: Sedentary behavior
SLD: Steatotic liver disease
STROBE: Strengthening the reporting of observational studies in epidemiology
T2DM: Type 2 diabetes mellitus
TG: Triglycerides
WC: Waist circumference
γGT: Gamma-glutamyl transferase
