# Supplementary material for: Associations of activity, sedentary and sleep behaviors with prevalent steatotic liver disease in middle-aged and older adults: the ELSA-Brasil study
Source: J Act Sedentary Sleep Behav. 2024 Jul 3;3:16. doi: 10.1186/s44167-024-00055-7 (PMC11960374; doi:10.1186/s44167-024-00055-7)
Supplement: Supplementary file 1 [file 44167_2024_55_MOESM1_ESM.docx]

SUPPLEMENTARY MATERIAL

**ASSOCIATIONS OF ACTIVITY, SEDENTARY AND SLEEP BEHAVIORS WITH PREVALENT STEATOTIC LIVER DISEASE IN MIDDLE-AGED AND OLDER ADULTS: THE ELSA-BRASIL STUDY**

Danilo de Paula^1^, Natan Feter^1^, Rodrigo Citton Padilha dos Reis^1,2^, Rosane Harter Griep^3^ Bruce Bartholow Duncan^1,4^, Maria Inês Schmidt^1,4^

1-Postgraduate Program in Epidemiology, Universidade Federal do Rio Grande do Sul, Brazil

2- Department of Statistics, Universidade Federal do Rio Grande do Sul, Brazil

3- Laboratório de Educação em Ambiente e Saúde, Instituto Oswaldo Cruz, Fundação Oswaldo Cruz, Rio de Janeiro, Brazil

4- Center for Clinical Research, Hospital de Clínicas de Porto Alegre, Brazil

**Corresponding author:** Danilo de Paula danilodpsantos@gmail.com

[1. SUPPLEMENTARY METHODS 4](#_Toc169632793)

[*Compositional data analysis* 4](#_Toc169632794)

[*Definitions of steatotic liver disease for the sensitivity analyses* 6](#_Toc169632795)

[Metabolic dysfunction associated steatotic liver disease (MASLD) 6](#_Toc169632796)

[Non-alcoholic fatty liver disease (NAFLD) 6](#_Toc169632797)

[2. SUPPLEMENTARY FIGURES 8](#_Toc169632798)

[Supplementary Table 1 Metabolic characteristics according to the presence of steatotic liver disease. ELSA-Brasil study wave 3 (2017-1029), n = 8569 8](#_Toc169632799)

[Supplementary Table 2 Time spent on movement behaviors during the 24-hours ELSA-Brasil (2017-1029), n = 8569. 10](#_Toc169632800)

[Supplementary Figure 1 24-hour movement behaviors according to the presence of steatotic liver disease ELSA-Brasil (2017-1029), n = 8569. 11](#_Toc169632801)

[Supplementary Table 3 Association of movement behaviors with steatotic liver disease among short sleepers (< 7h/day) by sex. ELSA-Brasil (2017-2019), n = 2.966 12](#_Toc169632802)

[Supplementary Table 4 Association of movement behaviors with steatotic liver disease among non-short sleepers (≥ 7h/day) by sex. ELSA-Brasil (2017-2019), n = 5.603 13](#_Toc169632803)

[Supplementary Figure 2 Dose-response associations of total activity volume with prevalent steatotic liver disease according to sleep duration and sex. ELSA-Brasil (2017-2019), n = 8569 14](#_Toc169632804)

[Supplementary Figure 3 Dose-response associations of moderate and vigorous physical activity with prevalent steatotic liver disease according to sleep duration and sex. ELSA-Brasil (2017-2019), n = 8569 16](#_Toc169632805)

[Supplementary Figure 4 Association of exchanging movement behaviors with the prevalence of steatotic liver disease in short sleepers (<7 h/day) by sex. ELSA-Brasil study (2017-2019), n = 2966 18](#_Toc169632806)

[Supplementary Figure 5 Association of exchanging movement behaviors with the prevalence of steatotic liver disease in non-short sleepers (≥7 h/day) by sex. ELSA-Brasil (2017-2019), n = 5603 19](#_Toc169632807)

[Supplementary Table 5 Association of movement behaviors and prevalent MASLD. ELSA-Brasil (2017-2019), n = 8569. 20](#_Toc169632808)

[Supplementary Figure 6 Dose-response associations of Total activity volume and MVPA with prevalent MASLD according to sleep duration. ELSA-Brasil (2017-2019), n = 8569 21](#_Toc169632809)

[Supplementary Figure 7 Association of exchanging movement behaviors with prevalent MASLD according to sleep duration. ELSA-Brasil (2017-2019), n = 8569. 23](#_Toc169632810)

[Supplementary Table 7 Association of the device measured movement behaviors and prevalence of MASLD. ELSA-Brasil participants without excessive alcohol consumption (2017-2019) n = 7.627 24](#_Toc169632811)

[Supplementary Figure 8 Dose-response associations of movement behaviors with the prevalence of MASLD ELSA-Brasil study participants without excessive alcohol consumption (2017-2019) n = 7.627 25](#_Toc169632812)

[Supplementary Figure 9 Association of exchanging movement behaviors with prevalence of MASLD, ELSA-Brasil study participants without excessive alcohol consumption (2017-2019), n = 7627 27](#_Toc169632813)

[3. STROBE STATEMENT - Checklist of items that should be included in reports of cross-sectional studies 28](#_Toc169632814)

# SUPPLEMENTARY METHODS

***Compositional data analysis***

Compositional data carry important information in the proportions between their components rather than in the absolute values of its components. This assumption holds when investigating the association of movement behaviors (i.e. physical activity and sedentary behavior) with health outcomes. Using adequate methods to examine the associations of daily movement behaviors with health outcomes is important because 1) time in each day is finite and constrained to 24 hours; 2) the engagement in movement behaviors is mutually exclusive, meaning that an individual can only perform one movement behavior at a time; 3) classic statistical models have limited potential to deal with the high collinearity between movement behaviors in a day.

Therefore, to evaluate if the composition of movement behaviors in the 24 hours was associated with the prevalence of SLD, we used compositional data analysis (CoDA).^1–4^ In CoDA, the duration of behaviors is constrained in a natural space called simplex, and their value is represented by coordinates that better picture the compositional data distribution. The procedures were conducted following previous research using CoDA methods in movement behaviors epidemiology and are described below.

We calculated the geometric mean of each movement behavior duration (and adjusted them to 24 hours) for the total sample and each subgroup because it better represents the central tendency of compositional data. Then, we calculated *isometric log ratios* (*ilr*) of the behaviors using the R statistical software version 4.2.1. (R Core Team, 2021, The R Foundation for Statistical Computing, Vienna, Austria). Epicoda package *transform_comp* function.^5,6^ The *isometric log-ratio* transformation generates a set of coordinates and is obtained by sequentially partitioning one behavior against all the remaining behaviors. For example, 1^st,^ we inserted time spent in moderate and vigorous physical activity against time in light physical activity, sedentary behavior, and sleep, 2^nd^, light physical activity against sedentary behavior and sleep; and finally, sedentary behavior against sleep. We generated a set of three *ilr* coordinates for each participant (i) named z­_i1,_ z_i2,_ and z_i3_ that capture the distribution of all components of the 24h movement behaviors when combined. The example set for the relative contribution of MVPA is shown below, where *i* stands for a given observation (subject).

$$Z_{i1}= \sqrt{\frac{3}{4}}\ln\left( \frac{{MVPA}_{i}}{\sqrt[3]{{LPA}_{i}*{SB}_{i}*{Sleep}_{i}}} \right)$$

$$Z_{i2}= \sqrt{\frac{2}{3}}\ln\left( \frac{LPAi}{\sqrt{{SB}_{i}*{Sleep}_{i}}} \right)$$

$$Z_{i3}= \sqrt{\frac{1}{2}}\ln\left( \frac{{SB}_{i}}{\sqrt{{Sleep}_{i}}} \right)$$

To investigate if the composition of the whole movement behaviors was associated with the prevalence of SLD, we assessed the maximum likelihood statistics of the robust Poisson models, including the set of 3 *ilr* as predictor variables. Models were progressively adjusted for the potential confounders named as models 1, 2a, and 2b in **the main text**. Furthermore, to interpret the results and estimate the association of reallocating time between behaviors with a change in prevalence of SLD, we used isotemporal substitution predictions based on the compositional models that had been previously built. This analysis was conducted by creating theoretical compositions derived from the geometric mean of the sample shown in **Supplementary Table 2** through pairwise substitutions of behaviors of 1 to 60 minutes a day. The theoretical compositions were then used to predict the expected prevalence ratios and their 95% confidence intervals relative to the specific group's geometric average of behaviors. To facilitate interpretation, results were presented as pairwise reallocation plots. Reallocation plots should be interpreted as the expected change in the prevalence of SLD for the average individual in the sample.

***Definitions of steatotic liver disease for the sensitivity analyses***

### Metabolic dysfunction associated steatotic liver disease (MASLD)

MASLD was defined according to the consensus criteria^7^ by a Fatty Liver Index ≥60 plus metabolic dysfunction identified by:

A - Body Mass Index ≥ 25 kg/m^2^ OR Waist circumference > 94cm (males) / 80cm (females) OR ethnicity-adjusted equivalents

B – Fasting plasma glucose ≥ 100 mg/dL OR 2-hour 75grams post-load plasma glucose ≥140 mg/dL

C – Blood pressure ≥ 130/85 mmHg OR antihypertensive medication

D – Plasma triglycerides ≥150mg/dL OR lipid-lowering medication

E – Plasma HDL-cholesterol ≤40 mg/dL (males) / 50 mg/dL (females) OR lipid-lowering medication

### Non-alcoholic fatty liver disease (NAFLD)

MASLD was defined by a Fatty Liver Index ≥60 in the absence of excessive alcohol consumption, identified by the ingestion of 210 grams/week (males) / 140 grams/week (females).^8^

1. Dumuid D, Stanford TE, Martin-Fernández JA, Pedišić Ž, Maher CA, Lewis LK, et al. Compositional data analysis for physical activity, sedentary time and sleep research. Stat Methods Med Res. 2018 Dec 1;27(12):3726–38.

2. Dumuid D, Pedišić Ž, Stanford TE, Martín-Fernández JA, Hron K, Maher CA, et al. The compositional isotemporal substitution model: A method for estimating changes in a health outcome for reallocation of time between sleep, physical activity and sedentary behaviour. Stat Methods Med Res. 2019 Mar;28(3):846–57.

3. Chastin SFM, Palarea-Albaladejo J, Dontje ML, Skelton DA. Combined Effects of Time Spent in Physical Activity, Sedentary Behaviors and Sleep on Obesity and Cardio-Metabolic Health Markers: A Novel Compositional Data Analysis Approach. Devaney J, editor. PLoS ONE. 2015 Oct 13;10(10):e0139984.

4. Chastin SFM, McGregor DE, Biddle SJH, Cardon G, Chaput JP, Dall PM, et al. Striking the Right Balance: Evidence to Inform Combined Physical Activity and Sedentary Behavior Recommendations. Journal of Physical Activity and Health. 2021 May 14;18(6):631–7.

5. OxWearables/epicoda [Internet]. Oxford Wearables Group; 2024 Available from: https://github.com/OxWearables/epicoda

6. Walmsley R, Chan S, Smith-Byrne K, Ramakrishnan R, Woodward M, Rahimi K, et al. Reallocation of time between device-measured movement behaviours and risk of incident cardiovascular disease. Br J Sports Med. 2021 Sep 6;bjsports-2021-104050.

7. Rinella ME, Lazarus JV, Ratziu V, Francque SM, Sanyal AJ, Kanwal F, et al. A multi-society Delphi consensus statement on new fatty liver disease nomenclature. Hepatology [Internet]. 2023 Jun 24 [cited 2023 Jul 24];Publish Ahead of Print. Available from: https://journals.lww.com/10.1097/HEP.0000000000000520

8. Chalasani N, Younossi Z, Lavine JE, Charlton M, Cusi K, Rinella M, et al. The diagnosis and management of nonalcoholic fatty liver disease: Practice guidance from the American Association for the Study of Liver Diseases. Hepatology. 2018 Jan;67(1):328–57.

# SUPPLEMENTARY FIGURES

**Supplementary Table 1 Metabolic characteristics according to the presence of steatotic liver disease. ELSA-Brasil study wave 3 (2017-1029), n = 8569**

|  | **SLD  n=3764** | **No SLD  n=4805** | **Overall  n=8569** |
| --- | --- | --- | --- |
| **Metabolic dysfunction - number** | 4.02 (1.42) | 1.93 (1.31) | 2.85 (1.71) |
| **Glucose metabolism** |  |  |  |
| Fasting plasma glucose - mg/dL | 121 (40.7) | 104 (23.3) | 112 (33.1) |
| 2 hours glucose 75g OTTG - mg/dL | 140 (51.2) | 114 (35.4) | 125 (44.3) |
| HbA1C (%) | 6.00 (1.31) | 5.56 (0.81) | 5.75 (1.08) |
| Fasting insulin - mcUI/mL | 18.9 (15.7) | 9.83 (11.2) | 13.8 (14.1) |
| pre-diabetes - n | 3268 (86.9%) | 3143 (65.4%) | 6411 (74.8%) |
| Diabetes - n | 1389 (36.9%) | 730 (15.2%) | 2119 (24.7%) |
| **Lipids metabolism** |  |  |  |
| Total cholesterol - mg/dL | 200 (42.6) | 196 (38.1) | 198 (40.2) |
| Triglycerides - mg/dL | 181 (134) | 108 (45.6) | 140 (102) |
| Hypertrigliceridemia - n | 1948 (51.8%) | 716 (14.9%) | 2664 (31.1%) |
| HDL - mg/dL | 48.8 (13.3) | 59.9 (16.2) | 55.0 (15.9) |
| Low HDL - n(%) | 1571 (41.7%) | 923 (19.2%) | 2494 (29.1%) |
| LDL – mg/dL | 115 (36.6) | 114 (34.2) | 115 (35.3) |
| **Blood pressure** |  |  |  |
| Systolic blood pressure - mmHg | 126 (16.0) | 119 (16.3) | 122 (16.5) |
| Diastolyc blood pressure - mmHg | 79.2 (9.67) | 73.4 (9.36) | 75.9 (9.91) |
| High blood pressure – n | 1575 (41.8%) | 1169 (24.3%) | 2744 (32.0%) |
| **Body composition** |  |  |  |
| Body mass index - kg/m² | 31.4 (4.32) | 24.9 (2.89) | 27.8 (4.82) |
| High body mass index – n | 3631 (96.5%) | 2392 (49.8%) | 6023 (70.3%) |
| Waist circumference – cm | 106 (9.80) | 87.5 (8.33) | 95.6 (12.8) |
| High Waist circumference – n | 3046 (81.0%) | 1365 (28.4%) | 4411 (51.5%) |
| Body fat - % | 37.7 (8.50) | 31.7 (8.50) | 34.3 (9.01) |
| **Liver disease markers** |  |  |  |
| C reactive protein - mg/L | 4.06 (5.93) | 2.45 (4.97) | 3.16 (5.47) |
| Aspartate aminotransferase (AST) - U/L | 22.9 (13.3) | 19.8 (6.99) | 21.2 (10.3) |
| Alanine transaminase (ALT) - U/L | 26.8 (18.2) | 18.6 (9.11) | 22.2 (14.5) |
| Gamma-glutamyl transferase (GGT) -U/L | 55.4 (88.5) | 25.8 (19.3) | 38.8 (62.2) |
| Fatty liver index – score | 80.8 (11.6) | 30.0 (16.4) | 52.3 (29.1) |
| **Use of medication** |  |  |  |
| Blood pressure medication - n | 1928 (51.4%) | 1422 (29.7%) | 3350 (39.2%) |
| Glucose lowering medication – n | 955 (25.4%) | 502 (10.4%) | 1457 (17.0%) |
| Use of insulin – n | 6 (0.16%) | 4 (0.08%) | 10 (0.12%) |
| Lipid lowering medication – n | 1092 (29.0%) | 1146 (23.8%) | 2238 (26.1%) |

SLD = Metabolic dysfunction associated steatotic liver disease; OTTG = oral glucose tolerance test; HbA1C = glycated hemoglobin; HDL = high density lipoprotein, LDL = low density lipoprotein

**Supplementary Table 2 Time spent on movement behaviors during the 24-hours ELSA-Brasil (2017-1029), n = 8569.**

|  | MVPA | LPA | SB | Sleep^a^ |
| --- | --- | --- | --- | --- |
| **Arithmethic mean**  **minutes (SD)** |  |  |  |  |
| Overall | 47.4 (25.3) | 207 (68.4) | 737 (99.8) | 448 (71.6) |
| Sleeps <7 hrs/day | 51.6 (26.1) | 216 (68.4) | 795 (87.4) | 377 (35.3) |
| Sleeps ≥7 hrs/day | 45.2 (24.5) | 202 (67.9) | 707 (92.3) | 486 (55.7) |
| **Geometric mean minutes** |  |  |  |  |
| Overall | 40.53 | 200.26 | 746.33 | 452.88 |
| Sleeps <7 hrs/day | 44.92 | 209.01 | 803.83 | 382.24 |
| Sleeps ≥7 hrs/day | 38.19 | 194.81 | 714.03 | 492.97 |

MVPA = moderate and vigorous physical activity; LPA = light physical activity; SB = sedentary behavior; a = as measured by sleep diary in the same week of accelerometer data collection. Geometric means were closed to 24 hours

**Supplementary Figure 1 24-hour movement behaviors according to the presence of steatotic liver disease ELSA-Brasil (2017-1029), n = 8569.**

**
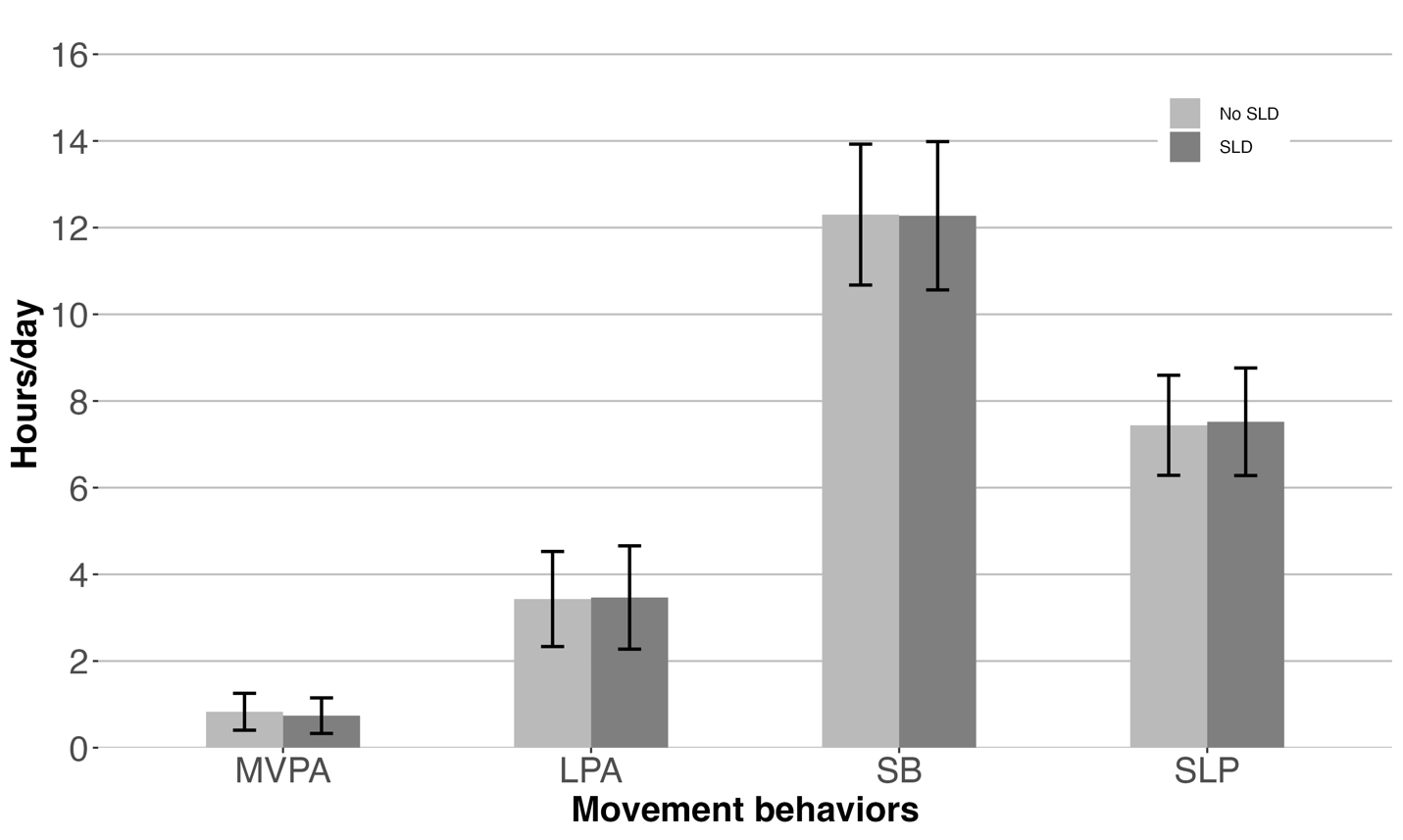
**

SLD = steatotic liver disease; MVPA = moderate and vigorous physical activity; LPA = light physical activity; SB = sedentary behavior, SLP = Sleep

**Supplementary Table 3 Association of movement behaviors with steatotic liver disease among short sleepers (< 7h/day) by sex. ELSA-Brasil (2017-2019), n = 2.966**

| **Model** | **Overall activity**  **1 m*g*/day**  *PR (95% CI)* | **MVPA**  **30 minutes/day**  *PR (95% CI)* | **LPA**  **30 minutes/day**  *PR (95% CI)* | **SB**  **30 minutes/day**  *PR (95% CI)* | **Sleep**  **30 minutes/day**  *PR (95% CI)* |
| --- | --- | --- | --- | --- | --- |
|  |  | ***Female*** *n = 1510* | | |  |
| **M1** | 0.96 (0.93; 0.99) | 0.83 (0.73; 0.94) | 1.01 (0.97; 1.05) | 1.00 (0.97; 1.03) | 1.04 (0.96; 1.12) |
| **M2a** | 0.99 (0.96; 1.03) | 0.97 (0.86; 1.10) | 1.01 (0.98; 1.05) | 0.99 (0.96; 1.02) | 1.01 (0.94; 1.09) |
| **M2b** | 1.00 (0.97; 1.03) | 0.96 (0.84; 1.09) | 1.02 (0.98; 1.06) | 0.99 (0.96; 1.02) | 1.01 (0.94; 1.09) |
|  |  |  |  |  |  |
|  |  |  | ***Male***  *n = 1456* |  |  |
| **M1** | 0.96 (0.94; 0.99) | 0.87 (0.80; 0.95) | 0.99 (0.96; 1.03) | 1.02 (0.99; 1.05) | 0.99 (0.93; 1.05) |
| **M2a** | 0.99 (0.97; 1.01) | 0.95 (0.87; 1.04) | 1.01 (0.98; 1.04) | 1.00 (0.98; 1.03) | 0.99 (0.93; 1.05) |
| **M2b** | 0.99 (0.97; 1.01) | 0.95 (0.87; 1.04) | 1.02 (0.98; 1.05) | 1.00 (0.97; 1.03) | 0.97 (0.91; 1.03) |

SLD = Steatotic liver disease; PR = Prevalence ratios, CI = confidence interval, MVPA = moderate to vigorous physical activity, LPA = light physical activity, SB = Sedentary behavior. Robust variance Poisson regression was adjusted progressively as listed: Model 1 = adjusted for study center, age, race/color, income, degree of schooling, smoking, alcohol consumption, diabetes, hypertension, and daily energy intake; Model 2a = Model 1 plus body mass index; Model 2b = Model 1 plus % body fat.

**Supplementary Table 4 Association of movement behaviors with steatotic liver disease among non-short sleepers (≥ 7h/day) by sex. ELSA-Brasil (2017-2019), n = 5.603**

| **Model** | **Total activity volume**  **1 m*g*/day**  *PR (95% CI)* | **MVPA**  **30 minutes/day**  *PR (95% CI)* | **LPA**  **30 minutes/day**  *PR (95% CI)* | **SB**  **30 minutes/day**  *PR (95% CI)* | **Sleep**  **30 minutes/day**  *PR (95% CI)* |
| --- | --- | --- | --- | --- | --- |
|  |  | ***Female*** *n = 3.265* | | |  |
| **M1** | 0.94 (0.92; 0.96) | 0.77 (0.70; 0.84) | 0.99 (0.96; 1.01) | 1.01 (0.99; 1.03) | 1.02 (0.99; 1.05) |
| **M2a** | 0.99 (0.97; 1.01) | 0.94 (0.87; 1.03) | 1.01 (0.99; 1.04) | 1.00 (0.98; 1.01) | 1.00 (0.97; 1.03) |
| **M2b** | 0.98 (0.96; 1.01) | 0.92 (0.85; 1.01) | 1.01 (0.98; 1.03) | 1.00 (0.98; 1.02) | 1.00 (0.97; 1.03) |
|  |  |  | ***Male***  *n = 2.338* |  |  |
| **M1** | 0.97 (0.95; 0.99) | 0.87 (0.81; 0.94) | 1.00 (0.98; 1.02) | 1.01 (0.99; 1.03) | 1.01 (0.98; 1.04) |
| **M2a** | 0.99 (0.97; 1.01) | 0.95 (0.88; 1.02) | 1.00 (0.98; 1.02) | 1.00 (0.98; 1.02) | 1.01 (0.98; 1.04) |
| **M2b** | 0.99 (0.97; 1.01) | 0.94 (0.87; 1.02) | 1.00 (0.98; 1.03) | 1.00 (0.98; 1.02) | 1.00 (0.97; 1.04) |

SLD = steatotic liver disease; PR = Prevalence ratios, CI = confidence interval; MVPA = moderate to vigorous physical activity; LPA = light physical activity; SB = Sedentary behavior. Robust variance Poisson regression was adjusted progressively as listed: Model 1 = adjusted for study center, age, race/color, income, degree of schooling, smoking, alcohol consumption, diabetes, hypertension, and daily energy intake; Model 2a = Model 1 plus body mass index; Model 2b = Model 1 plus % body fat.

**Supplementary Figure 2 Dose-response associations of total activity volume with prevalent steatotic liver disease according to sleep duration and sex. ELSA-Brasil (2017-2019), n = 8569**


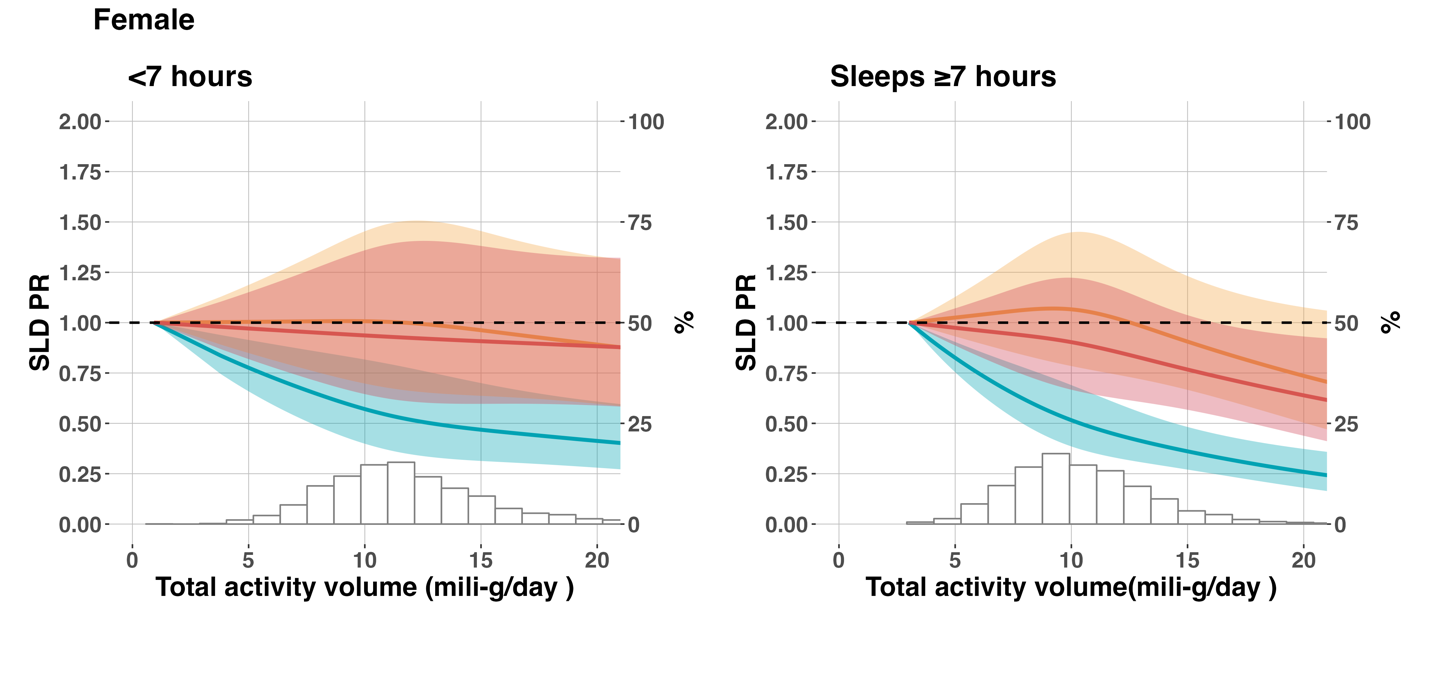

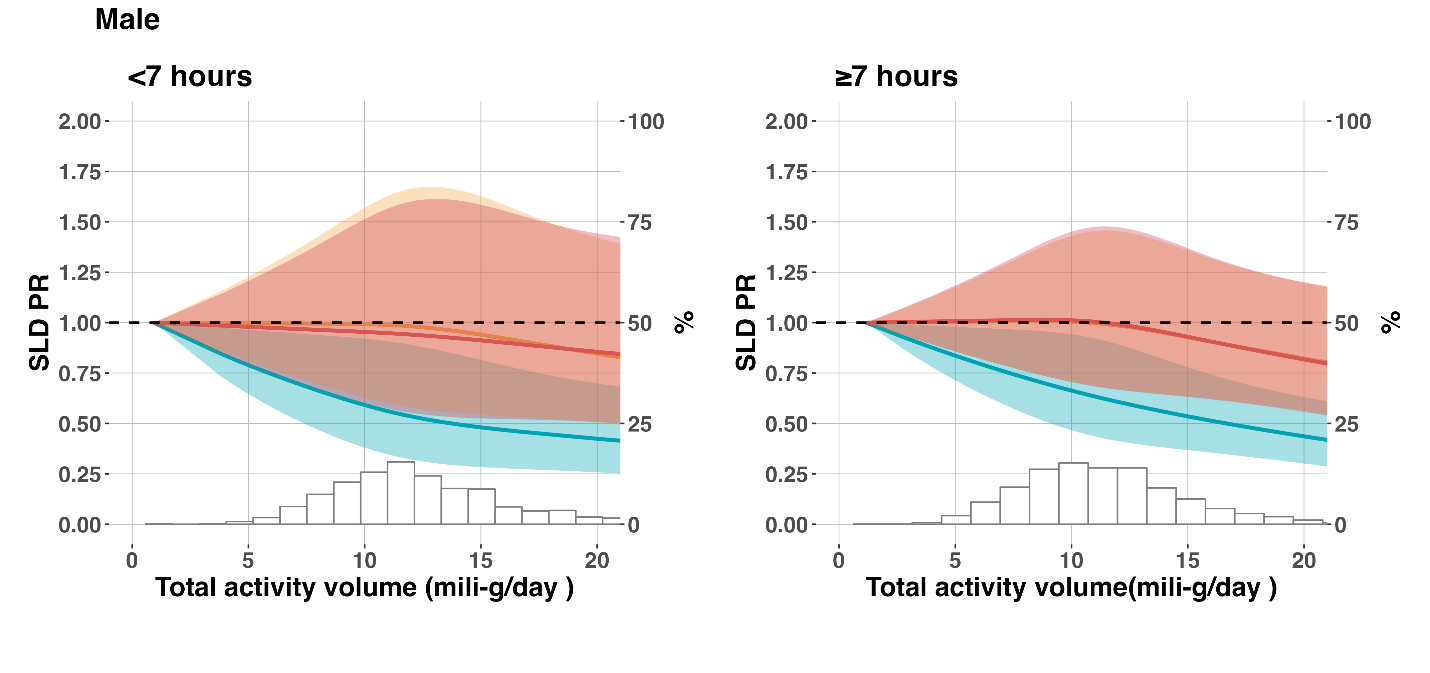

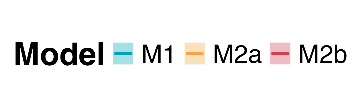


Prevalence ratios were estimated with Poisson regression models using restricted cubic splines with knots placed at the 10^th^, 50^th^, and 90^th^ percentiles of the exposure and adjusted for – M1 (blue): study center, sex, age, race/color, income, degree of schooling, smoking, alcohol consumption, and daily energy intake; M2a (yellow): M1 and further adjustment for body mass index. M2b (red): M1 and further adjustment for % body fat. All the splines use the lowest observed exposure level as the reference level. Continuous lines are the point estimates of prevalence ratios (PR) across the spectra of exposure, and the colored hatched area is the 95% confidence interval. The histograms show the distribution of the sample on the exposure spectra, with the right vertical axis showing the percentage of the study sample. SLD = steatotic liver disease; PR = prevalence ratios;

**Supplementary Figure 3 Dose-response associations of moderate and vigorous physical activity with prevalent steatotic liver disease according to sleep duration and sex. ELSA-Brasil (2017-2019), n = 8569**


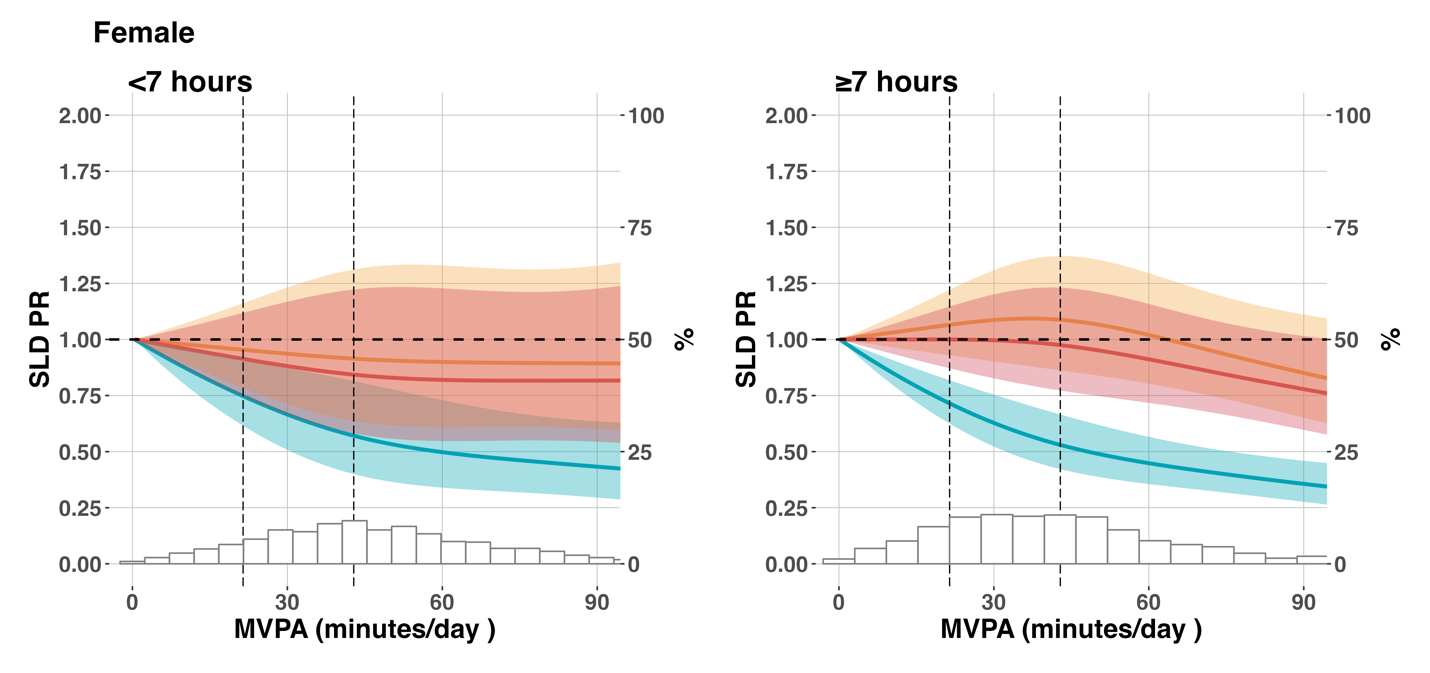

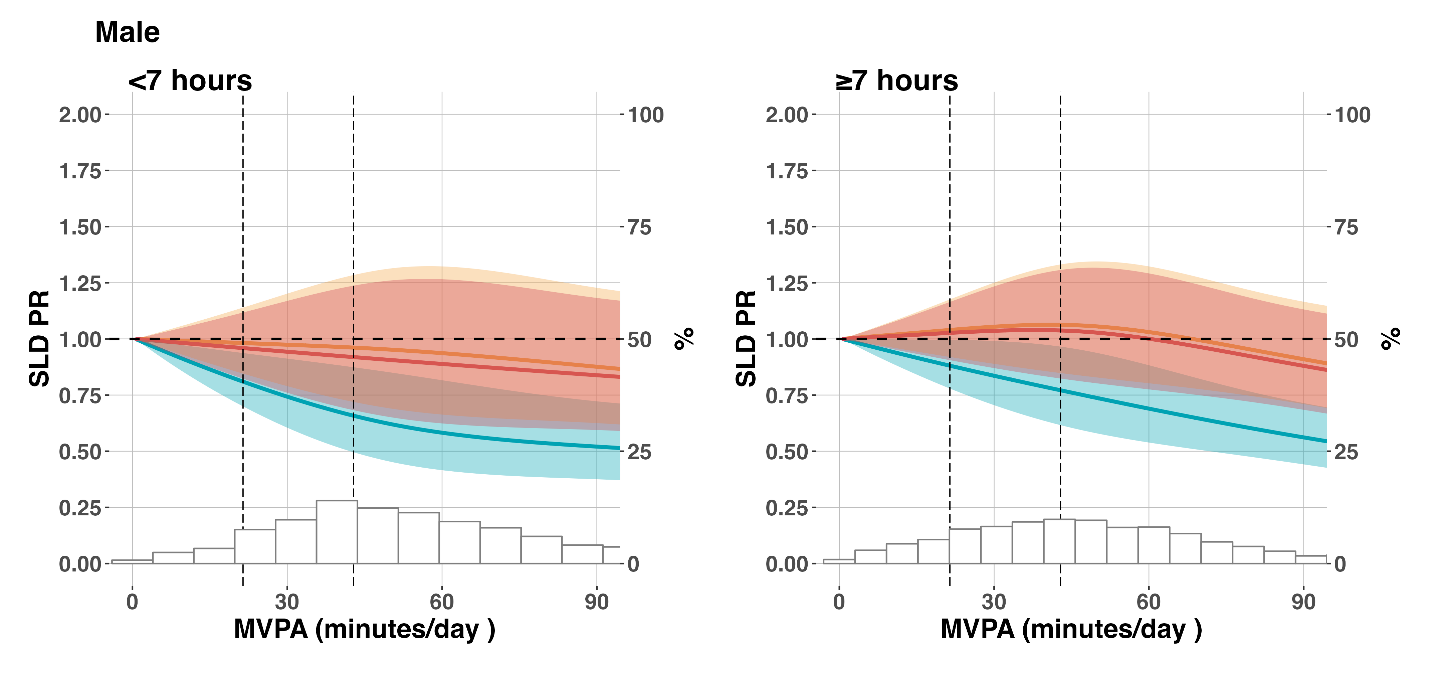

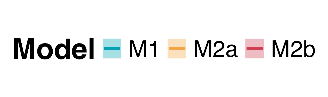


Prevalence ratios were estimated with Poisson regression models using restricted cubic splines with knots placed at 10^th^, 50^th^, and 90^th^ percentiles of the exposure and adjusted for – M1 (blue): study center, age, race/color, income, degree of schooling, smoking, alcohol consumption, diabetes, hypertension, and daily energy intake; M2a (yellow): M1 and further adjustment for body mass index. M2b (red): M1 and further adjustment for % body fat. All the splines use the lowest observed exposure level as the reference level. Continuous lines are the point estimates of prevalence ratios (PR) across the spectra of exposure, and the colored hatched area is the 95% confidence interval. The histograms show the distribution of the sample on the exposure spectra, with the right vertical axis showing the percentage of the study sample. SLD = Steatotic liver disease; PR = prevalence ratios; MVPA = moderate-and-vigorous physical activity

**Supplementary Figure 4 Association of exchanging movement behaviors with the prevalence of steatotic liver disease in short sleepers (<7 h/day) by sex. ELSA-Brasil study (2017-2019), n = 2966**


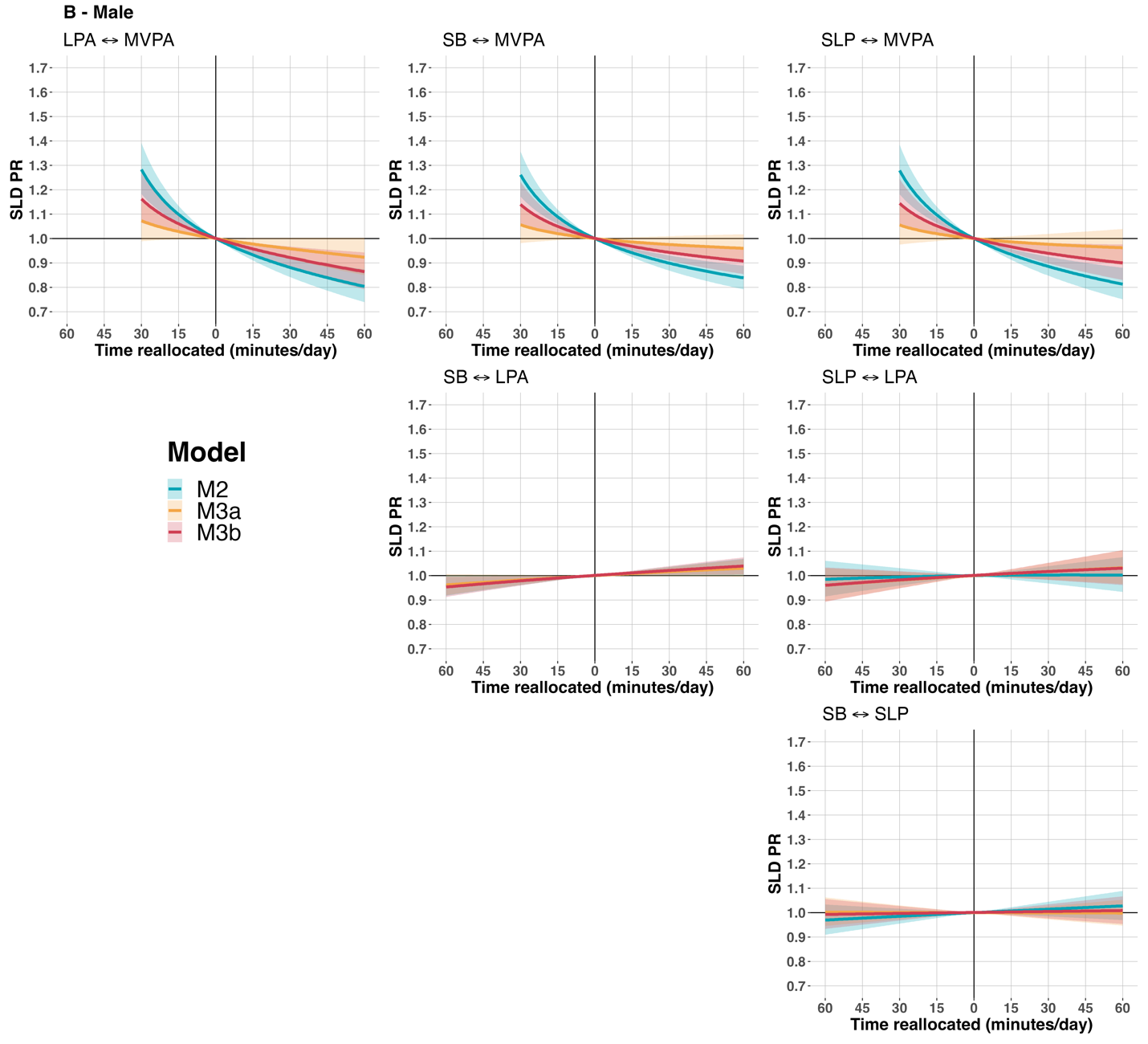

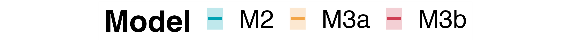

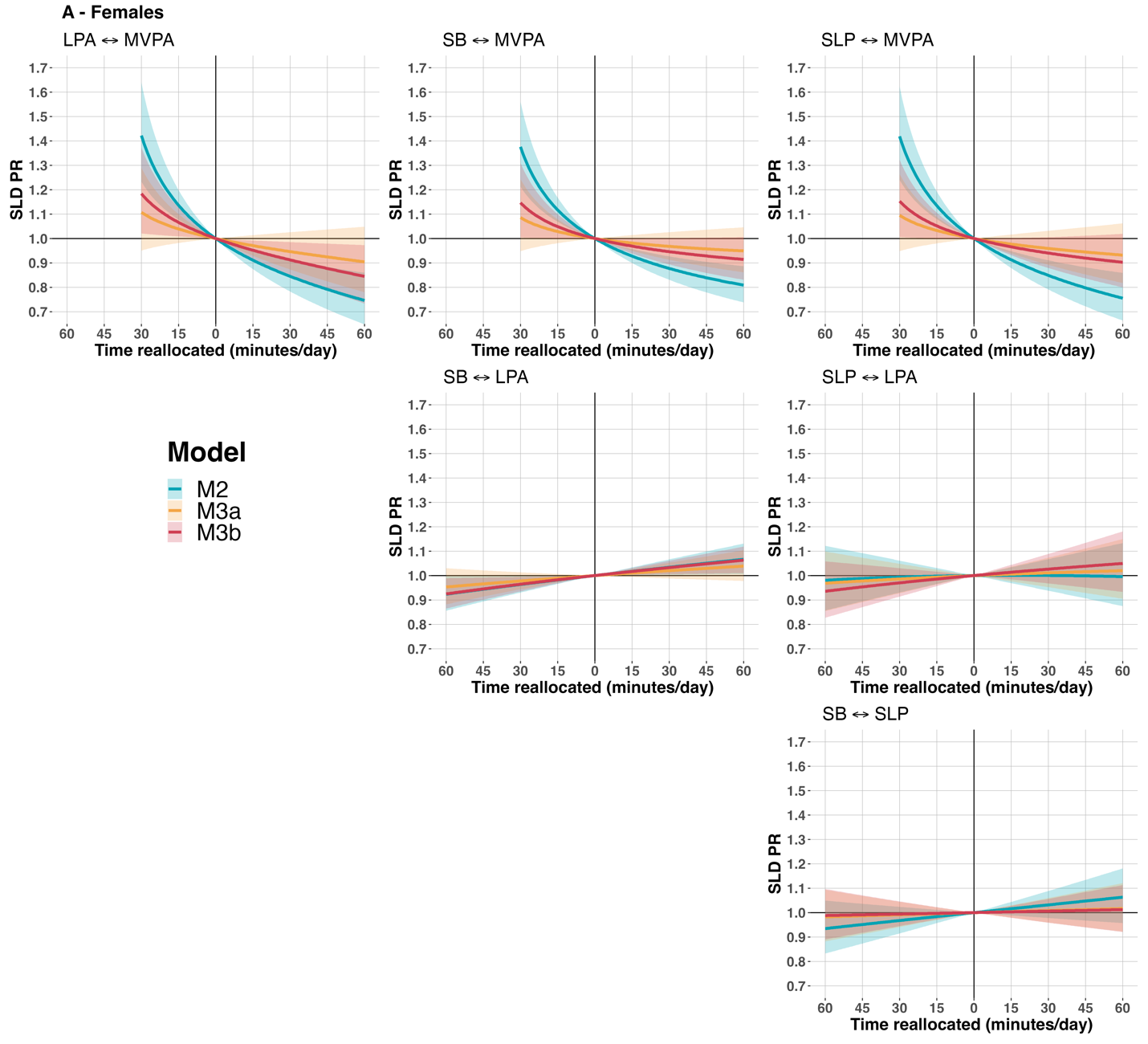

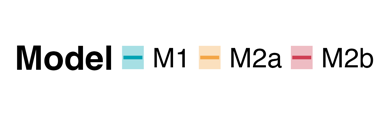


Plots show the predicted prevalence rate ratios of SLD resulting from reallocating time between movement behaviors using compositional isotemporal substitution Poisson models with robust variance. The plots show the expected prevalence rate ratio of exchanging behaviors using the geometric mean of behaviors of the group as the reference – A Females: MVPA: 41.7 minutes/day, LPA: 206.8 minutes/day, SB: 810 minutes/day, SLP: 381.6minutes/day; B Males: MVPA: 48.5 minutes/day, LPA: 211.3 minutes/day, SB: 797.4 minutes/day, SLP: 382.8 minutes/day. The two behaviors that are not shown in each plot have their values fixed at the geometric mean for the group of analysis. SLD = Steatotic liver disease; MVPA = moderate and vigorous physical activity; LPA = light physical activity; SB = sedentary behavior. Models were adjusted for – M1 (blue): study center, sex, age, race/color, income, degree of schooling, smoking, alcohol consumption, and daily energy intake; M2a (yellow): M1 and further adjustment for body mass index. M2b (red): M1 and further adjustment for % body fat.

**Supplementary Figure 5 Association of exchanging movement behaviors with the prevalence of steatotic liver disease in non-short sleepers (≥7 h/day) by sex. ELSA-Brasil (2017-2019), n = 5603**


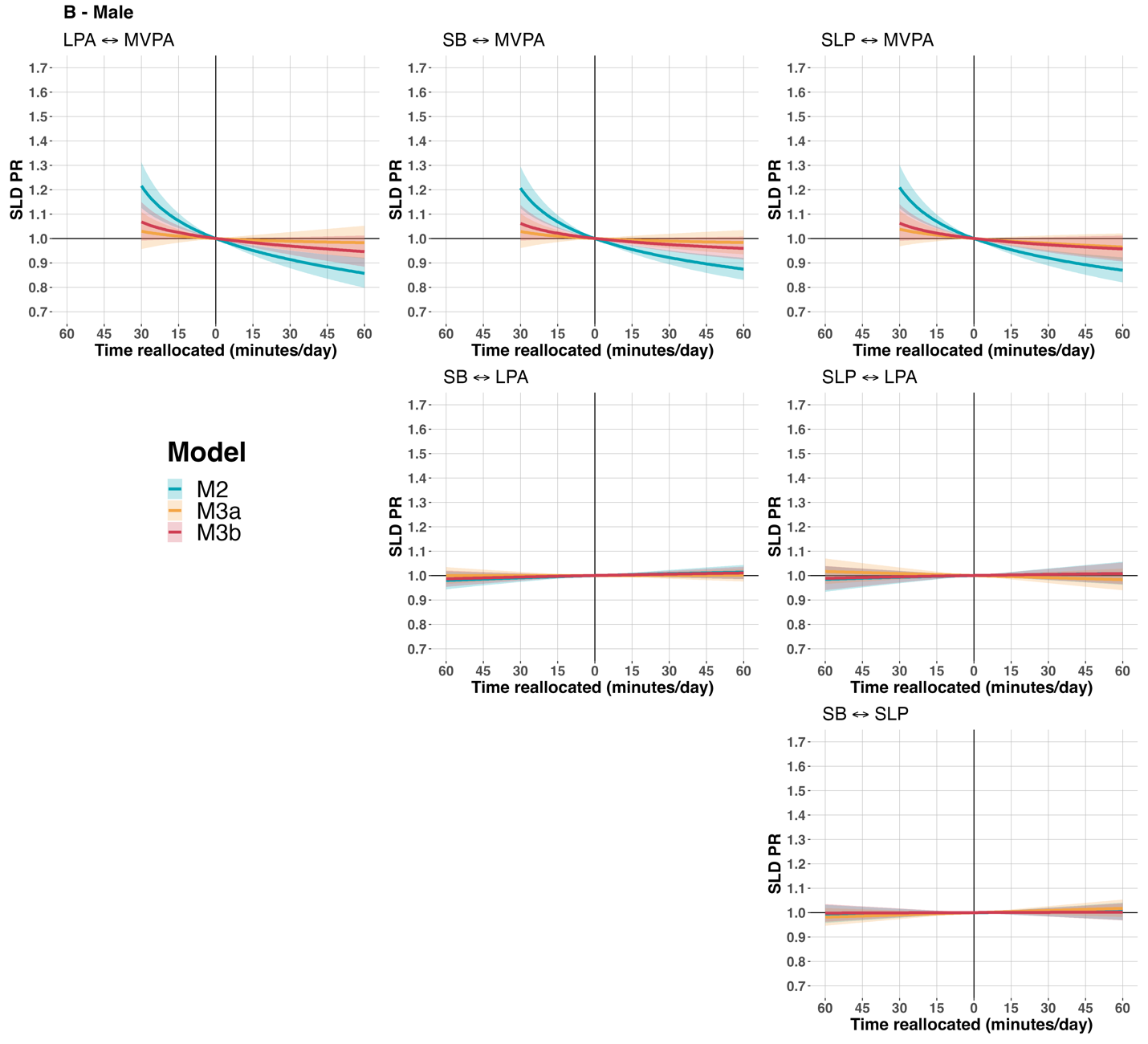

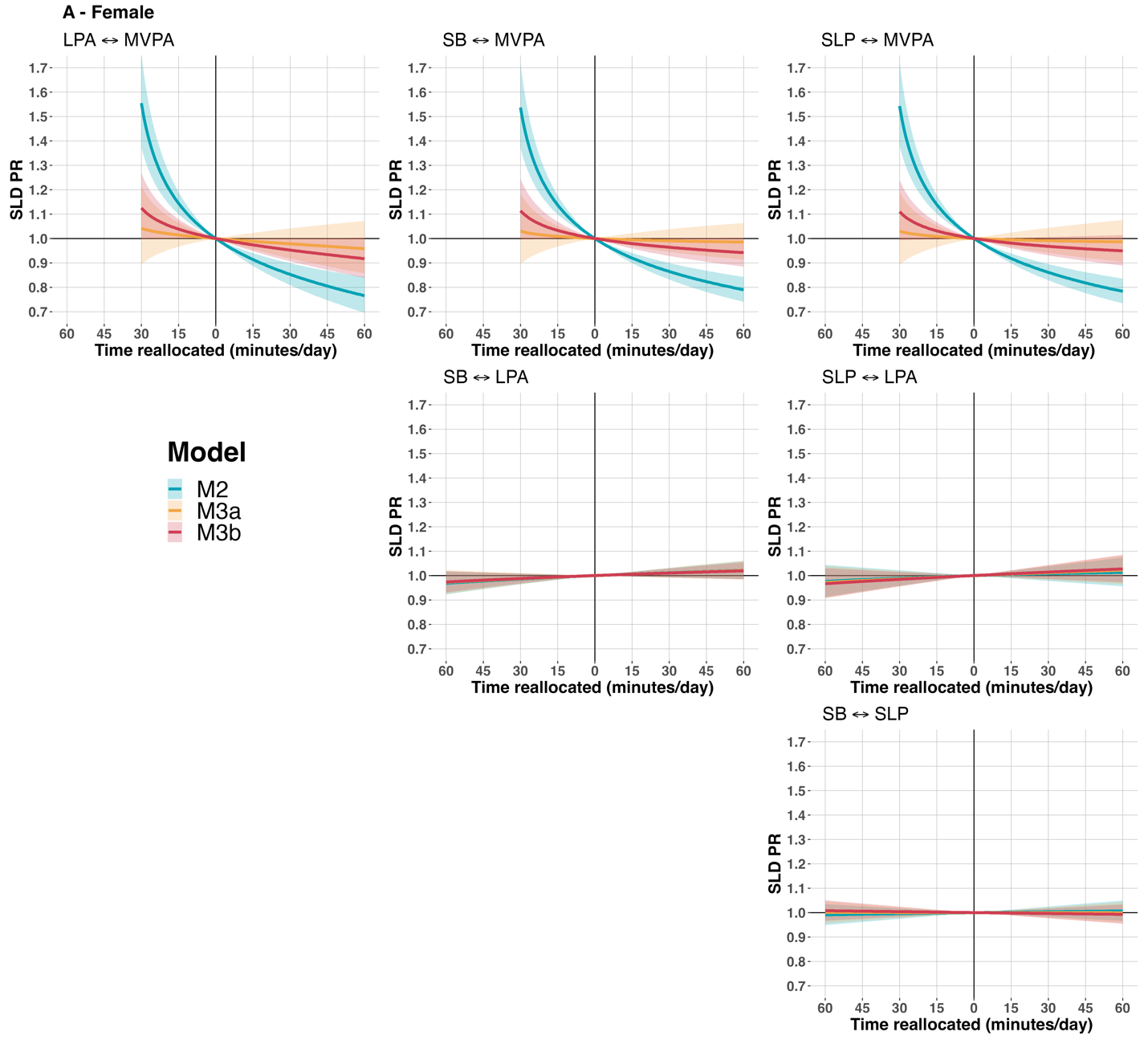

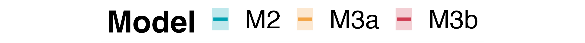

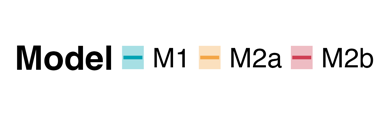


Plots show the predicted prevalence rate ratios of SLD resulting from reallocating time between movement behaviors using compositional isotemporal substitution Poisson models with robust variance. The plots show the expected prevalence rate ratio of exchanging behaviors using the geometric mean of behaviors of the group as the reference – Females: MVPA: 35.8 minutes/day, LPA: 193 minutes/day, SB: 717.5 minutes/day, SLP: 493.7 minutes/day; Males: MVPA: 41.8 minutes/day, LPA: 197.4 minutes/day, SB: 709 minutes/day, SLP: 491.9 minutes/day. The two behaviors that are not shown in each plot have their values fixed at the geometric mean for the group of analysis. SLD = Steatotic Liver Disease; MVPA = moderate and vigorous physical activity; LPA = light physical activity; SB = sedentary behavior. Models were adjusted for – M1 (blue): study center, sex, age, race/color, income, degree of schooling, smoking, alcohol consumption, and daily energy intake; M2a (yellow): M1 and further adjustment for body mass index. M2b (red): M1 and further adjustment for % body fat.

**Supplementary Table 5 Association of movement behaviors and prevalent MASLD. ELSA-Brasil (2017-2019), n = 8569.**

| **Model** | **Total activity volume**  **1 m*g*/day**  *PR (95% CI)* | **MVPA**  **30min/day**  *PR (95% CI)* | **LPA**  **30min/day**  *PR (95% CI)* | **SB**  **30min/day**  *PR (95% CI)* | **Sleep**  **30min/day**  *PR (95% CI)* |
| --- | --- | --- | --- | --- | --- |
|  |  |  | *Sleep <7 hours* |  |  |
| **M1** | 0.95 (0.94; 0.97) | 0.83 (0.77; 0.89) | 1.00 (0.97; 1.02) | 1.02 (1.00; 1.04) | 1.00 (0.95; 1.04) |
| **M2a** | 0.99 (0.97; 1.01) | 0.95 (0.88; 1.02) | 1.01 (0.99; 1.04) | 1.00 (0.98; 1.02) | 1.00 (0.95; 1.04) |
| **M2b** | 0.99 (0.97; 1.01) | 0.94 (0.87; 1.01) | 1.02 (1.00; 1.05) | 1.00 (0.98; 1.02) | 0.98 (0.94; 1.03) |
|  |  | *Sleep ≥7 hours* | | |  |
| **M1** | 0.95 (0.93; 0.96) | 0.78 (0.74; 0.83) | 0.99 (0.97; 1.01) | 1.01 (1.00; 1.03) | 1.02 (1.00; 1.04) |
| **M2a** | 0.99 (0.97; 1.00) | 0.94 (0.89; 0.99) | 1.00 (0.98; 1.02) | 1.00 (0.99; 1.01) | 1.01 (0.99; 1.03) |
| **M2b** | 0.98 (0.97; 1.00) | 0.91 (0.86; 0.96) | 1.00 (0.99; 1.02) | 1.00 (0.99; 1.02) | 1.01 (0.98; 1.03) |

MASLD = Metabolic dysfunction associated steatotic liver disease; PR = Prevalence rate ratios, CI = confidence interval, MVPA = moderate to vigorous physical activity, LPA = light physical activity, SB = Sedentary behavior. Robust variance Poisson regression was adjusted progressively as listed: Model 1 = adjusted for study center, age, sex, race/color, income, degree of schooling, smoking, alcohol consumption, diabetes, hypertension, and daily energy intake; Model 2a = Model 1 plus body mass index; Model 2b = Model 1 plus % body fat.

**Supplementary Figure 6 Dose-response associations of Total activity volume and MVPA with prevalent MASLD according to sleep duration. ELSA-Brasil (2017-2019), n = 8569**


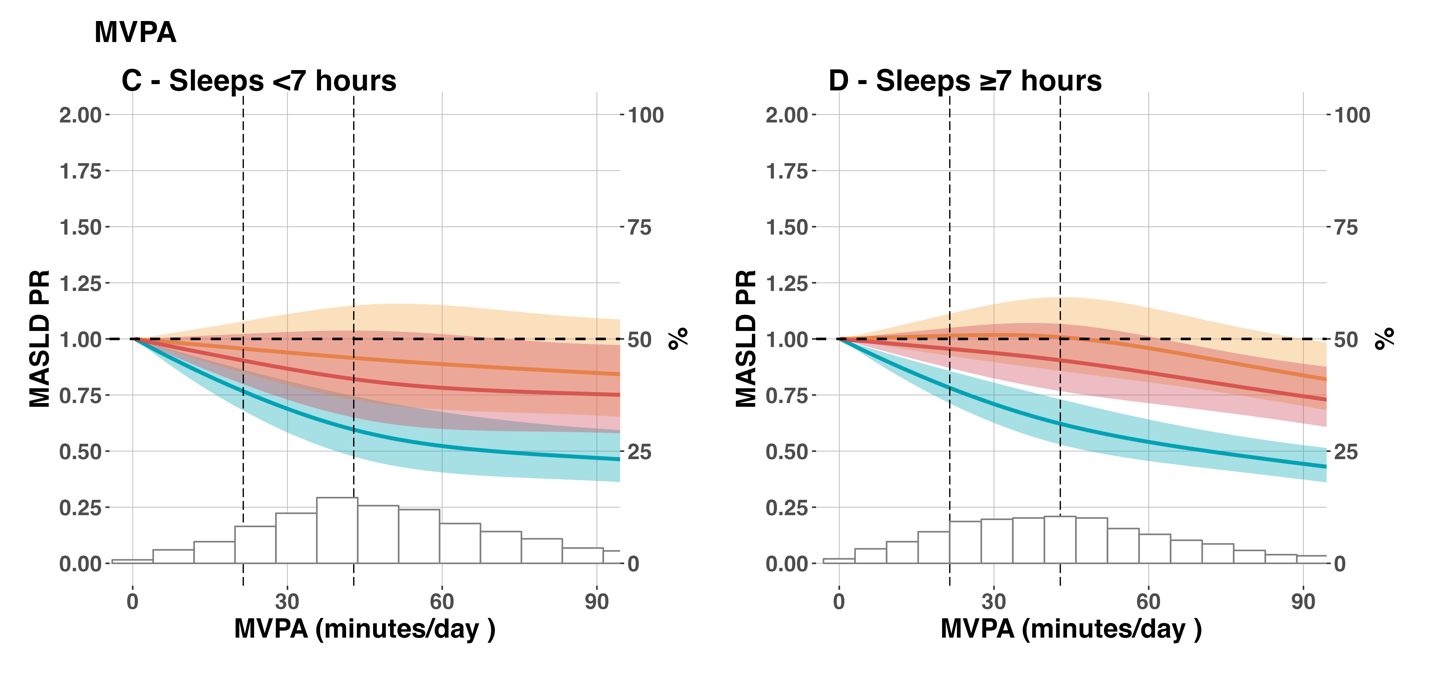

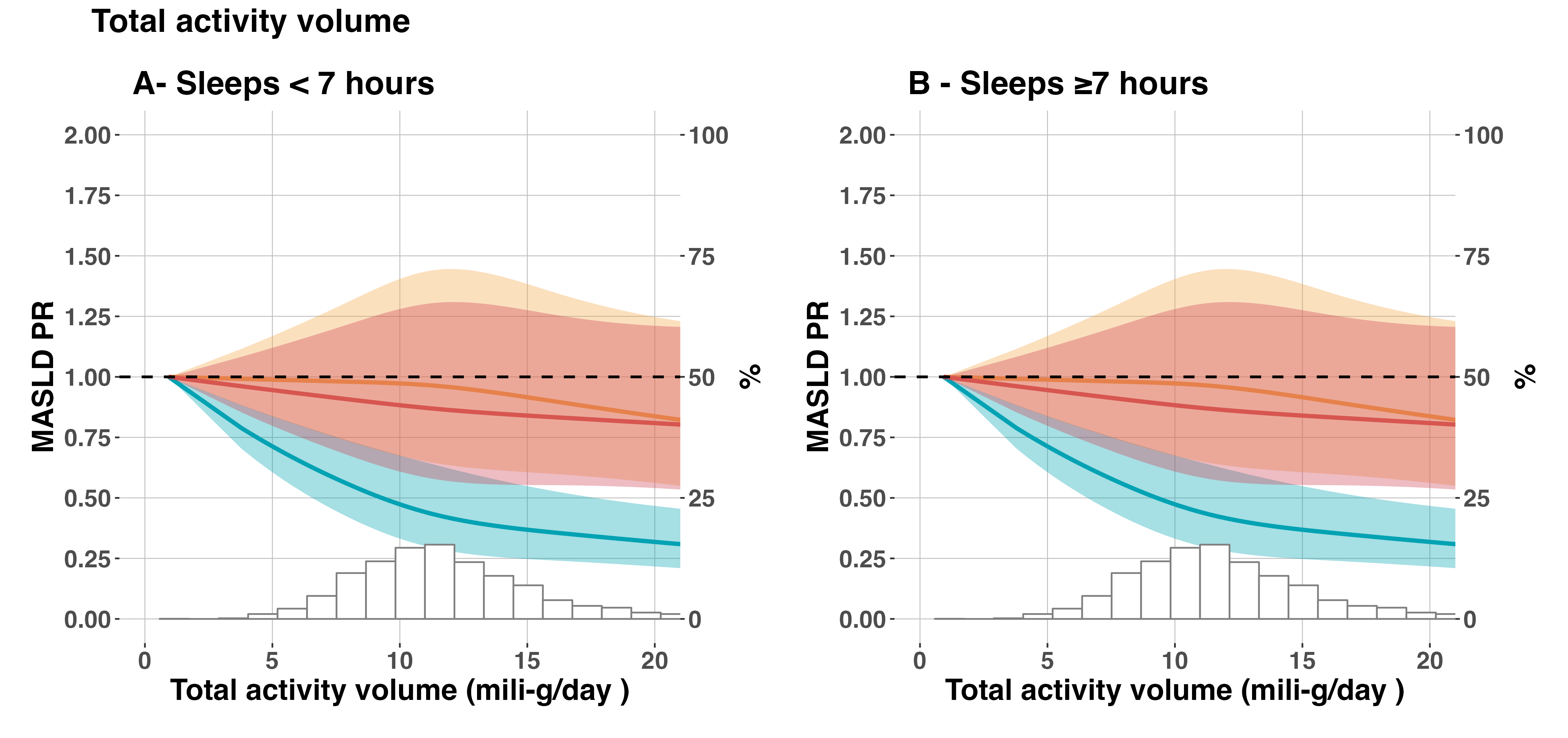

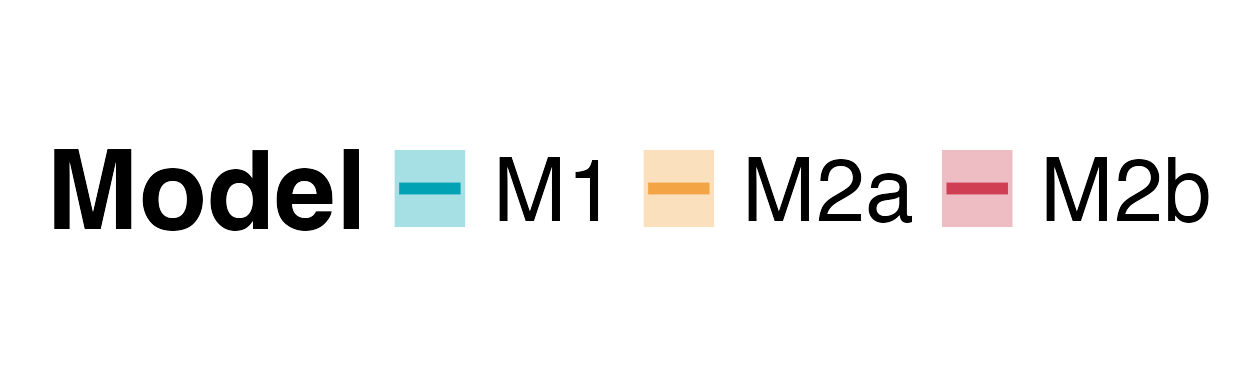


Prevalence Ratios (PA) for Metabolic dysfunction associated steatotic liver disease (MASLD) were estimated with Poisson regression models using restricted cubic splines with knots placed at 10^th^, 50^th^, and 90^th^ percentiles of the exposure and adjusted for – M1 (blue): study center, sex, age, race/color, income, degree of schooling, smoking, alcohol consumption, and total energy intake; M2a (yellow): M1 and further adjustment for body mass index. M2b (red): M1 and further adjustment for % body fat. All the splines use the lowest observed exposure level as the reference level. Continuous lines are the point estimates of PR across the spectra of exposure, and the colored hatched area is the 95% confidence interval. The histograms show the distribution of the sample on the exposure spectra, with the right vertical axis showing the percentage of the study sample. MVPA = moderate-and-vigorous physical activity

**Supplementary Figure 7 Association of exchanging movement behaviors with prevalent MASLD according to sleep duration. ELSA-Brasil (2017-2019), n = 8569.**


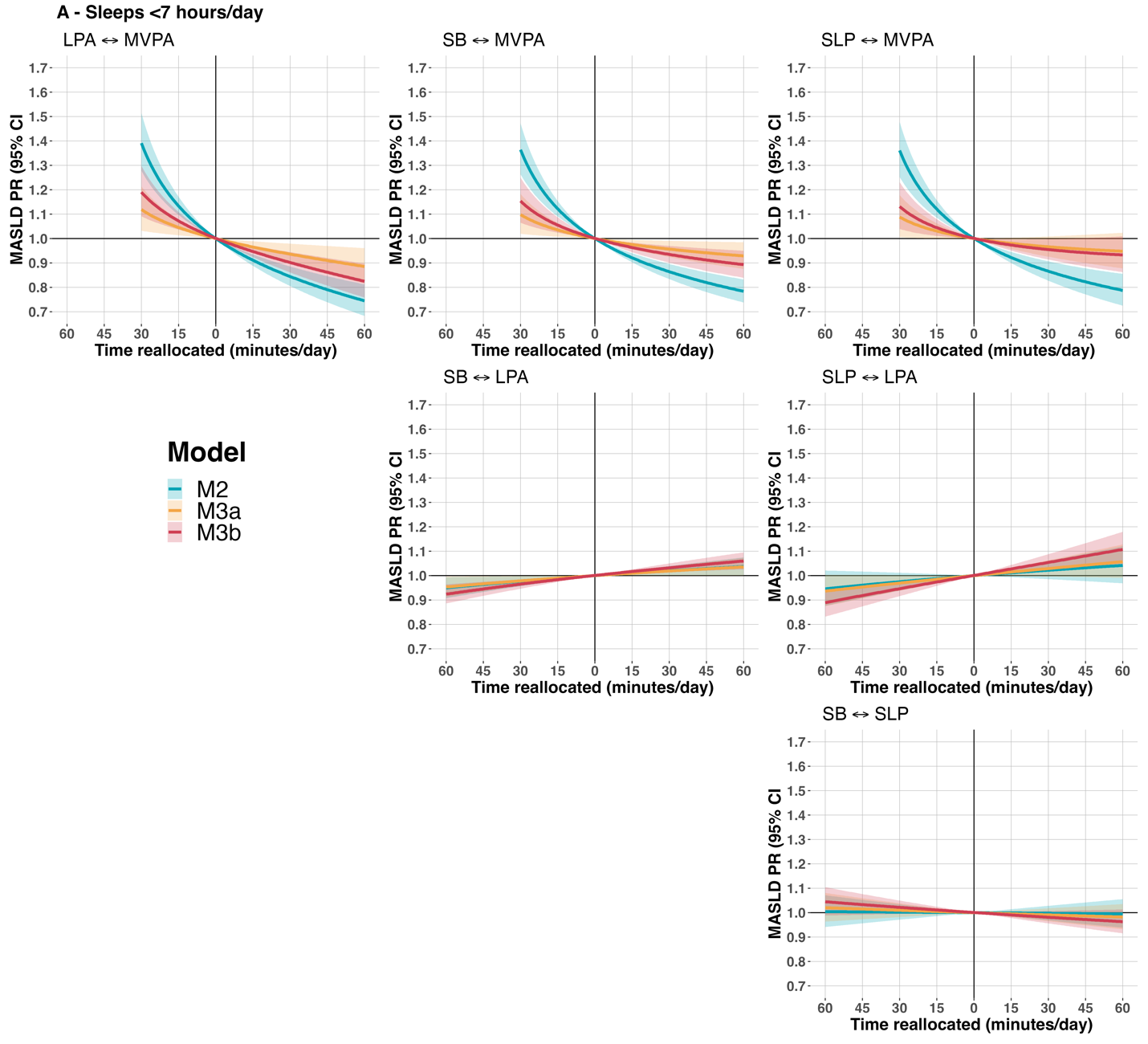

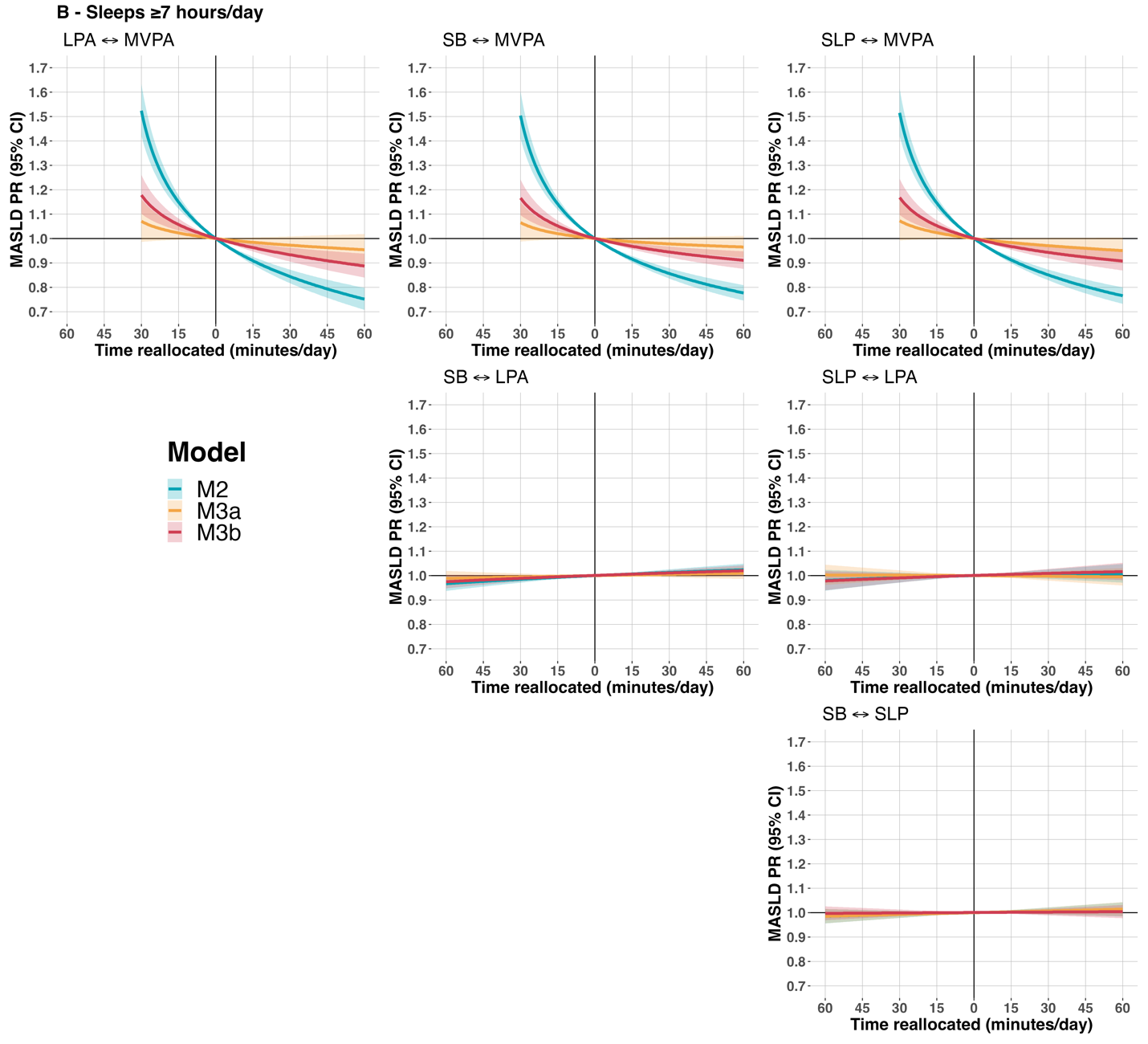

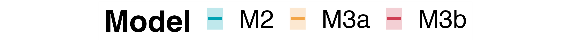

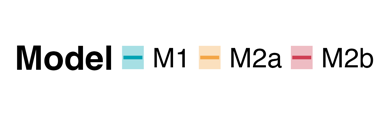


Plots show the predicted prevalence ratios (PR) of Metabolic dysfunction associated steatotic liver disease (MASLD) resulting from reallocating time between movement behaviors using compositional isotemporal substitution robust Poisson models. The vertical 0 line represents the mean of behaviors of the group as the reference – Sleep < 7 hours: MVPA: 44.9 minutes/day, LPA: 209 minutes/day, SB: 803.8 minutes/day, SLP: 382.2 minutes/day; Sleep ≥ 7 hours: MVPA: 38.2 minutes/day, LPA: 194.8 minutes/day, SB: 714 minutes/day, SLP: 493 minutes/day. The two behaviors that are not shown in each plot have their values fixed at the geometric mean for the group of analysis. MVPA = moderate and vigorous physical activity; LPA = light physical activity; SB = sedentary behavior. Models were adjusted as follows – Model 2 (M1, blue): study center, sex, age, race/color, income, degree of schooling, smoking, alcohol consumption, and total energy intake; Model 2a (M2a, yellow): M1 and further adjustment for body mass index. Model 2b (M2b, red): M1 and further adjustment for % body fat.

**Supplementary Table 7 Association of the device measured movement behaviors and prevalence of MASLD. ELSA-Brasil participants without excessive alcohol consumption (2017-2019) n = 7.627**

| **Model** | **Total activity volume**  **1 m*g*/day**  *PR (95% CI)* | **MVPA**  **30min/day**  *PR (95% CI)* | **LPA**  **30min/day**  *PR (95% CI)* | **SB**  **30min/day**  *PR (95% CI)* | **Sleep**  **30min/day**  *PR (95% CI)* |
| --- | --- | --- | --- | --- | --- |
|  |  |  | *Sleep <7 hours* |  |  |
| **M1** | 0.95 (0.94; 0.97) | 0.82 (0.76; 0.89) | 1.00 (0.97; 1.02) | 1.02 (1.00; 1.04) | 0.99 (0.94; 1.04) |
| **M2a** | 0.96 (0.95; 0.98) | 0.85 (0.79; 0.92) | 1.00 (0.98; 1.03) | 1.01 (0.99; 1.03) | 1.01 (0.96; 1.06) |
| **M2b** | 0.98 (0.96; 1.00) | 0.9 (0.83; 0.98) | 1.01 (0.98; 1.03) | 1.01 (0.98; 1.03) | 0.98 (0.94; 1.04) |
|  |  | *Sleep ≥7 hours* | | |  |
| **M1** | 0.95 (0.93; 0.96) | 0.79 (0.74; 0.83) | 0.99 (0.97; 1.01) | 1.02 (1.00; 1.03) | 1.02 (0.99; 1.04) |
| **M2a** | 0.95 (0.94; 0.97) | 0.82 (0.77; 0.87) | 0.99 (0.97; 1.01) | 1.01 (1.00; 1.03) | 1.01 (0.99; 1.03) |
| **M2b** | 0.98 (0.96; 0.99) | 0.89 (0.84; 0.95) | 11.00 (0.98; 1.01) | 1.01 (0.99; 1.02) | 1.00 (0.98; 1.02) |

MASLD = Metabolic dysfunction associated steatotic liver disease; PR = Prevalence rate ratios, CI = confidence interval, MVPA = moderate to vigorous physical activity, LPA = light physical activity, SB = Sedentary behavior. Robust variance Poisson regression was adjusted progressively as listed: Model 1 = adjusted for study center, age, sex, race/color, income, degree of schooling, smoking, alcohol consumption, diabetes, hypertension, and daily energy intake; Model 2a = Model 1 plus body mass index; Model 2b = Model 1 plus % body fat. Excessive alcohol consumption was defined as ≥210 grams/week for males and ≥140 grams/week for females.

**Supplementary Figure 8 Dose-response associations of movement behaviors with the prevalence of MASLD ELSA-Brasil study participants without excessive alcohol consumption (2017-2019) n = 7.627**


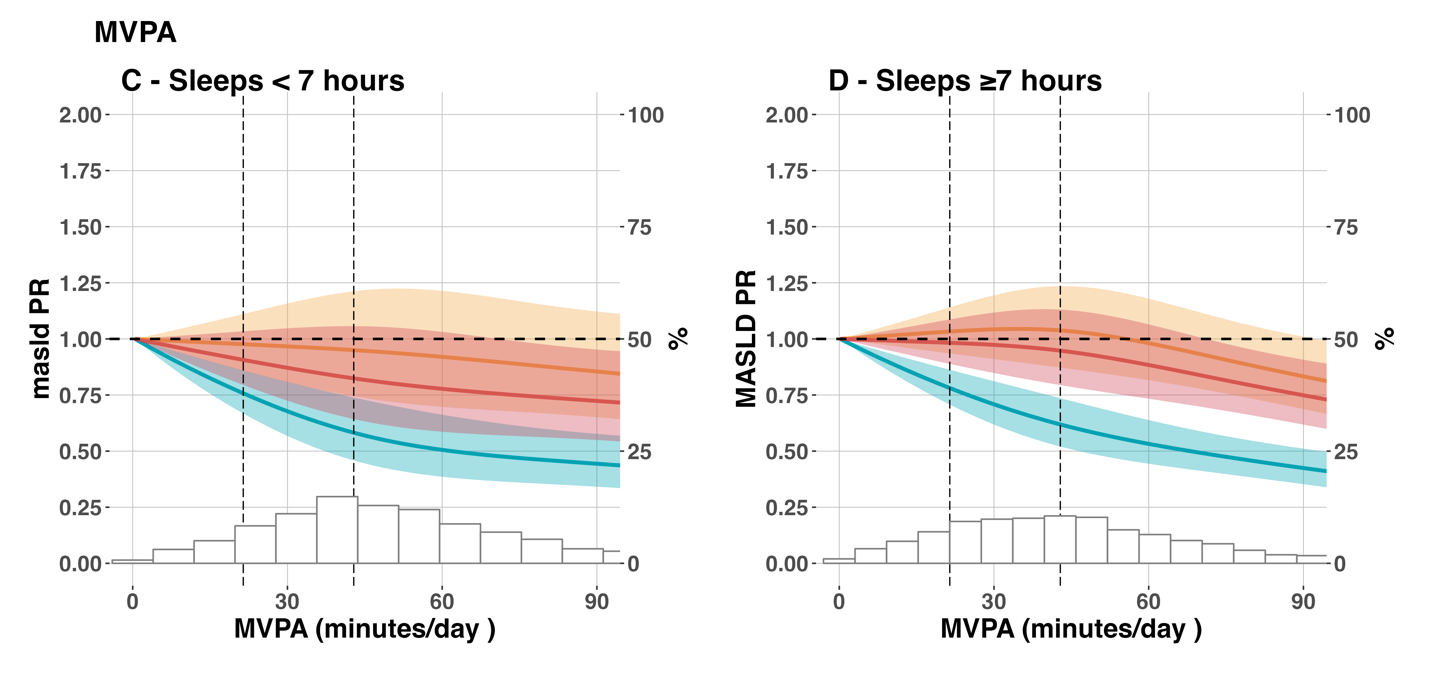

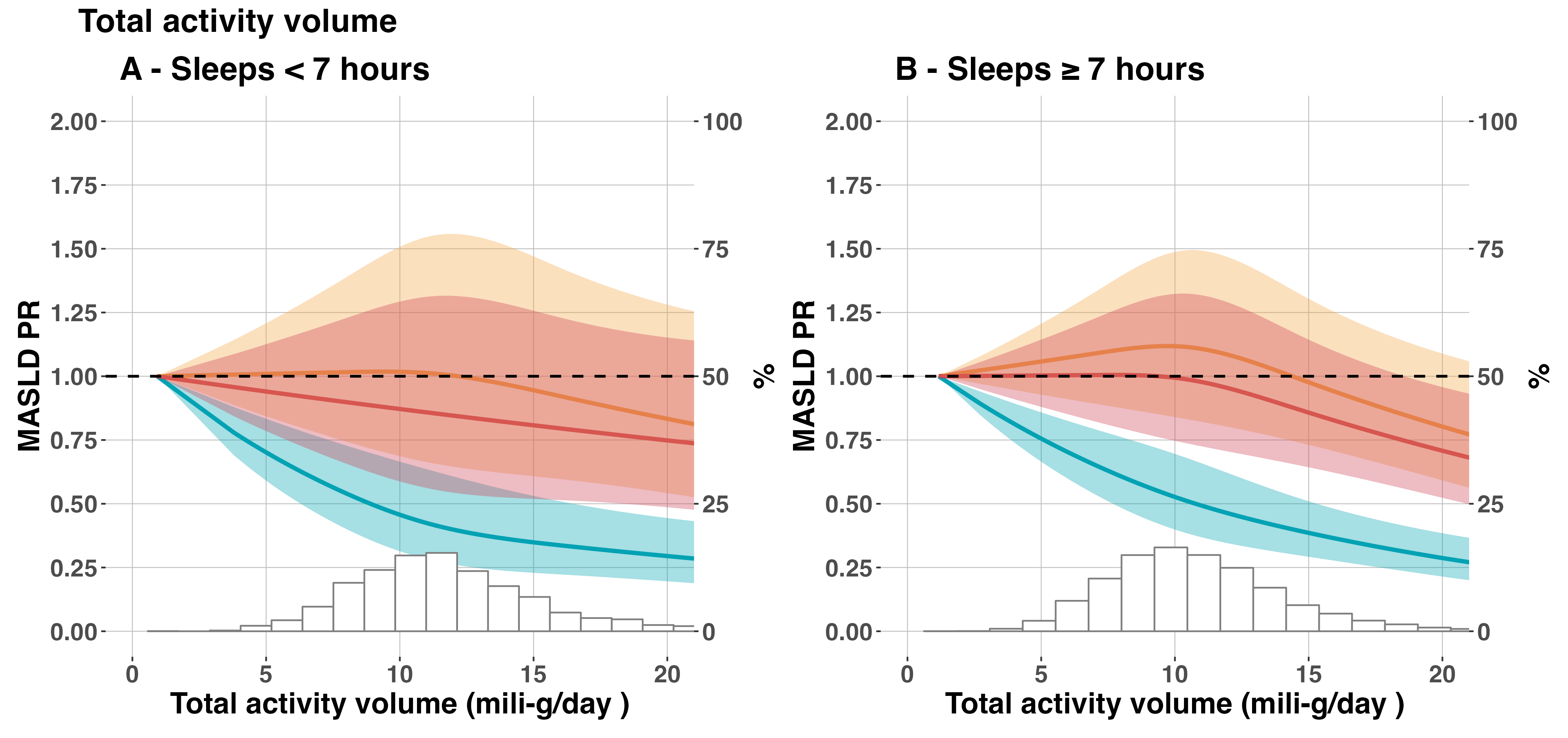

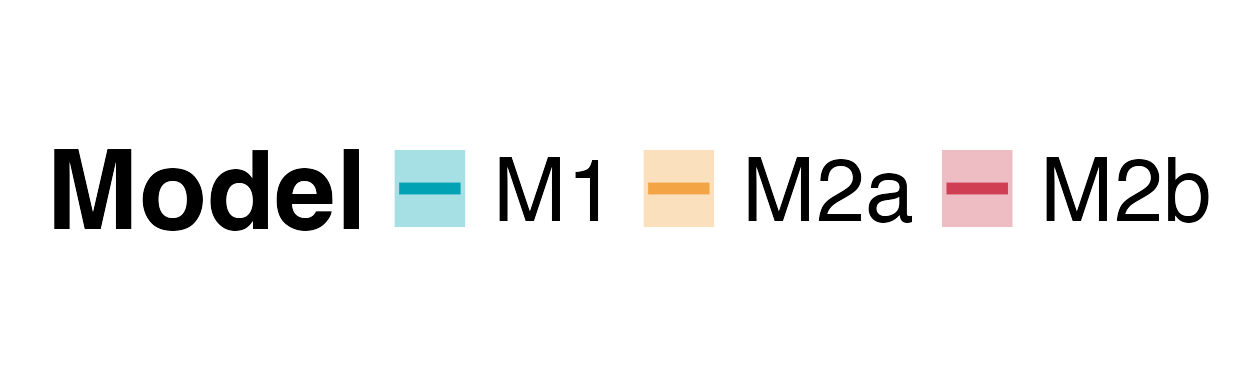


Prevalence ratios were estimated with Poisson regression models using restricted cubic splines with knots placed at 10^th^, 50^th^, and 90^th^ percentiles of the exposure and adjusted for – M1 (blue): study center, age, race/color, income, degree of schooling, smoking, alcohol consumption, and total energy intake; M2 (red): M1 and further adjustment for % body fat. All the splines use the lowest observed exposure level as the reference level. Continuous lines are the point estimates of prevalence ratios (PR) across the spectra of exposure, and the colored hatched area is the 95% confidence interval. The histograms show the distribution of the sample on the exposure spectra, with the right vertical axis showing the percentage of the study sample. MASLD = Metabolic dysfunction associated steatotic liver disease; PR = prevalence ratios; MVPA = moderate to vigorous physical activity. Excessive alcohol consumption was defined as ≥210 grams/week for males and ≥140 gr/week for females

**Supplementary Figure 9 Association of exchanging movement behaviors with prevalence of MASLD, ELSA-Brasil study participants without excessive alcohol consumption (2017-2019), n = 7627**


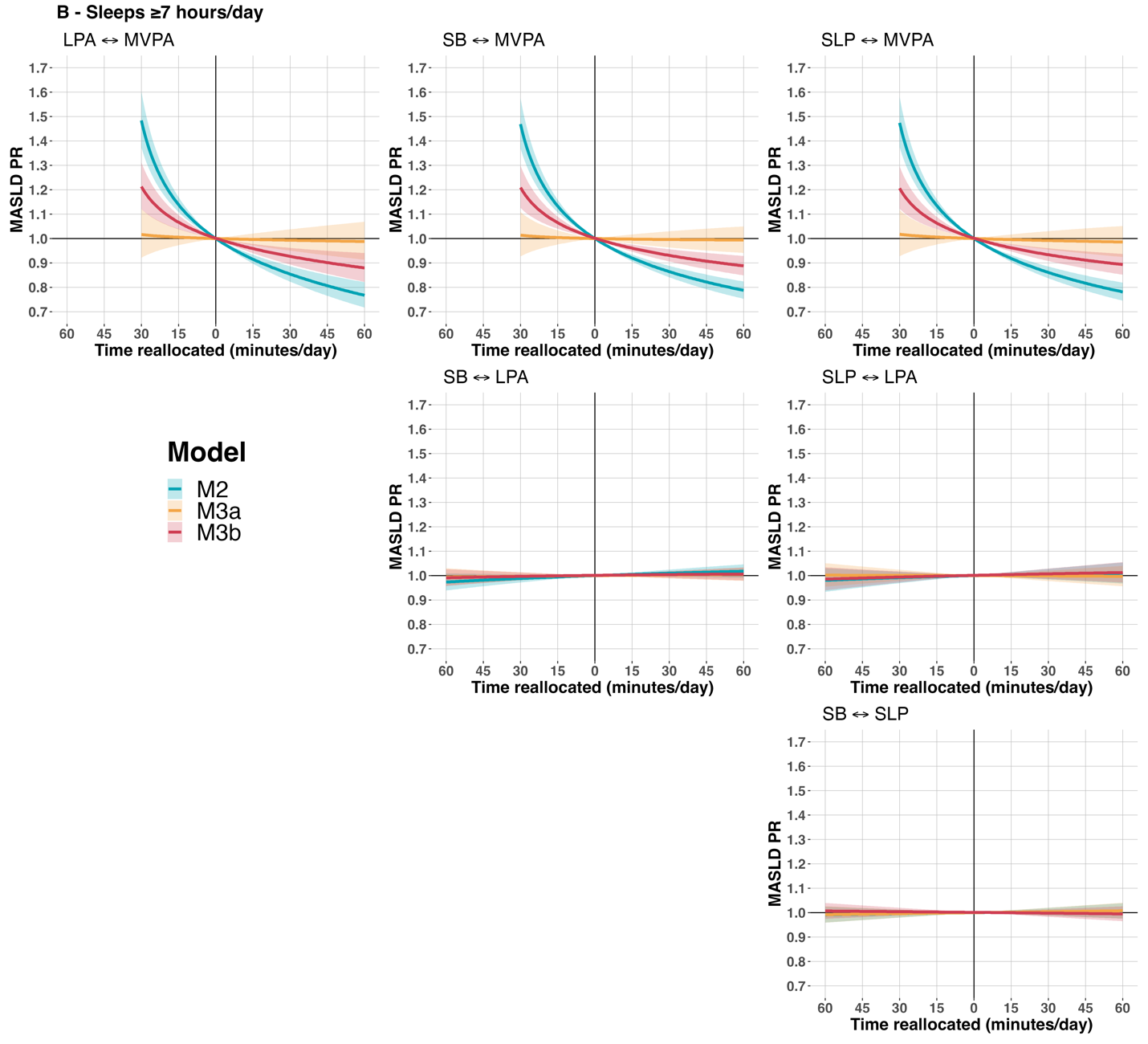

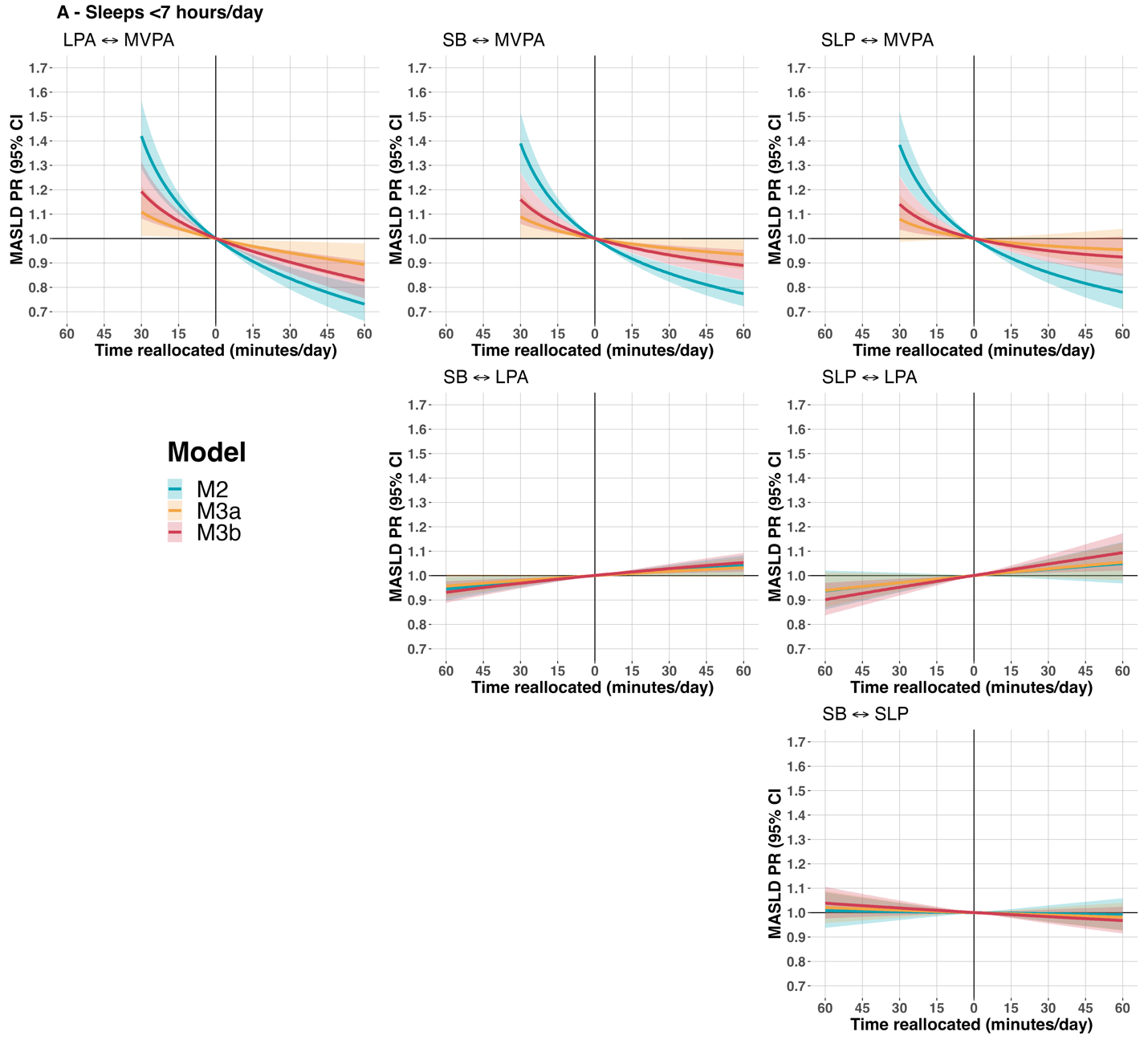

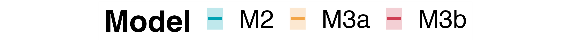

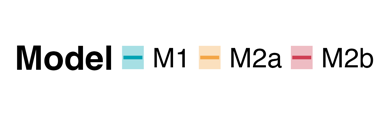


Plots show the predicted prevalence rate ratios of MASLD resulting from reallocating time between movement behaviors using compositional isotemporal substitution Poisson models with robust variance. The plots show the expected prevalence rate ratio of exchanging behaviors using the geometric mean of behaviors of the group as the reference – Sleeps < 7 hours MVPA: 44.4 minutes/day, LPA: 208.1 minutes/day, SB: 805 minutes/day, SLP: 382.1 minutes/day; Sleeps ≥ 7 hours MVPA: 38.1 minutes/day, LPA: 194.3 minutes/day, SB: 714.8 minutes/day, SLP: 492.7 minutes/day. The two behaviors that are not shown in each plot have their values fixed at the geometric mean for the group of analysis. MASLD = metabolic dysfunction associated steatotic liver disease; MVPA = moderate and vigorous physical activity; LPA = light physical activity; SB = sedentary behavior. Models were adjusted as follows: Model 1 = adjusted for study center, age, sex, race/color, income, degree of schooling, smoking, alcohol consumption, diabetes, hypertension, and daily energy intake; Model 2a = Model 1 plus body mass index; Model 2b = Model 1 plus % body fat. Excessive alcohol consumption was defined as ≥210 grams/week for males and ≥140 gr/week for females

# STROBE STATEMENT - Checklist of items that should be included in reports of *cross-sectional studies*

|  | Item No | Recommendation |  |
| --- | --- | --- | --- |
| **Title and abstract** | 1 | (*a*) Indicate the study’s design with a commonly used term in the title or the abstract | Yes, abstract methods |
|  |  | (*b*) Provide in the abstract an informative and balanced summary of what was done and what was found | Yes |
| Introduction | | |  |
| Background/rationale | 2 | Explain the scientific background and rationale for the investigation being reported | Pg 3 |
| Objectives | 3 | State specific objectives, including any prespecified hypotheses | Pg 3 |
| Methods | | |  |
| Study design | 4 | Present key elements of study design early in the paper | Pg 3-4 |
| Setting | 5 | Describe the setting, locations, and relevant dates, including periods of recruitment, exposure, follow-up, and data collection | Pg 3-4 |
| Participants | 6 | (*a*) Give the eligibility criteria, and the sources and methods of selection of participants | Pg 3-4 |
| Variables | 7 | Clearly define all outcomes, exposures, predictors, potential confounders, and effect modifiers. Give diagnostic criteria, if applicable | Pg 3-5 |
| Data sources/ measurement | 8* | For each variable of interest, give sources of data and details of methods of assessment (measurement). Describe comparability of assessment methods if there is more than one group | Pg 3-5 |
| Bias | 9 | Describe any efforts to address potential sources of bias | No |
| Study size | 10 | Explain how the study size was arrived at | No |
| Quantitative variables | 11 | Explain how quantitative variables were handled in the analyses. If applicable, describe which groupings were chosen and why | Pg 6 |
| Statistical methods | 12 | (*a*) Describe all statistical methods, including those used to control for confounding | Pg 6 |
|  |  | (*b*) Describe any methods used to examine subgroups and interactions | Pg 6 |
|  |  | (*c*) Explain how missing data were addressed | Pg 6/fig 1 |
|  |  | (*d*) If applicable, describe analytical methods taking account of sampling strategy | NA |
|  |  | (*e*) Describe any sensitivity analyses | Pg 6 |
| Results | | |  |
| Participants | 13* | (a) Report numbers of individuals at each stage of study—eg numbers potentially eligible, examined for eligibility, confirmed eligible, included in the study, completing follow-up, and analysed | Pg 7/Fig 1 |
|  |  | (b) Give reasons for non-participation at each stage | Pg 7/Fig 1 |
|  |  | (c) Consider use of a flow diagram | Figure 1 |
| Descriptive data | 14* | (a) Give characteristics of study participants (eg demographic, clinical, social) and information on exposures and potential confounders | Pg 7 and Table 1 |
|  |  | (b) Indicate number of participants with missing data for each variable of interest | No |
| Outcome data | 15* | Report numbers of outcome events or summary measures | Pg 7 |
| Main results | 16 | (*a*) Give unadjusted estimates and, if applicable, confounder-adjusted estimates and their precision (eg, 95% confidence interval). Make clear which confounders were adjusted for and why they were included | Pg 7-8/ Table 2 |
|  |  | (*b*) Report category boundaries when continuous variables were categorized | NA |
|  |  | (*c*) If relevant, consider translating estimates of relative risk into absolute risk for a meaningful time period | NA |
| Other analyses | 17 | Report other analyses done—eg analyses of subgroups and interactions, and sensitivity analyses | Pg 8-9/Supp results |
| Discussion | | |  |
| Key results | 18 | Summarise key results with reference to study objectives | Pg 9 |
| Limitations | 19 | Discuss limitations of the study, taking into account sources of potential bias or imprecision. Discuss both direction and magnitude of any potential bias | Pg 11-12 |
| Interpretation | 20 | Give a cautious overall interpretation of results considering objectives, limitations, multiplicity of analyses, results from similar studies, and other relevant evidence | Pg 09-12 |
| Generalisability | 21 | Discuss the generalisability (external validity) of the study results | Pg 12 |
| Other information | | |  |
| Funding | 22 | Give the source of funding and the role of the funders for the present study and, if applicable, for the original study on which the present article is based | Pg 13 |

*Give information separately for exposed and unexposed groups.

**Note:** An Explanation and Elaboration article discusses each checklist item and gives methodological background and published examples of transparent reporting. The STROBE checklist is best used in conjunction with this article (freely available on the Web sites of PLoS Medicine at http://www.plosmedicine.org/, Annals of Internal Medicine at http://www.annals.org/, and Epidemiology at http://www.epidem.com/). Information on the STROBE Initiative is available at www.strobe-statement.org.
